# A New Fluorine-18-Fluoroethyltyrosine Positron-Emission-Tomography/Magnetic-Resonance-Based Tumor Resection Plan Improved the Prognosis of Glioblastoma Patients. A Multicenter Validated Study

**DOI:** 10.34133/research.1335

**Published:** 2026-08-04

**Authors:** Ye Cheng, Xiaolong Wu, Bixiao Cui, Huantong Diao, Xiaohong Liang, Xu Chen, Feng Su, Siheng Liu, Bingyang Shan, Junfeng Lu, Haodan Dang, Chunlong Zhong, Wenhao Liu, Zihao Luan, Yue Du, Jie Tang, Jie Lu

**Affiliations:** ^1^ Department of Neurosurgery, Xuanwu Hospital, Capital Medical University and China International Neuroscience Institute, Beijing 100053, China.; ^2^ Department of Radiology and Nuclear Medicine, Xuanwu Hospital, Capital Medical University and Beijing Key Laboratory of Magnetic Resonance Imaging and Brain Informatics, Beijing 100053, China.; ^3^ Department of Neurosurgery, Tongji Hospital, Tongji Medical College of Huazhong University of Science & Technology, Wuhan 430030, China.; ^4^ College of Future Technology, Peking University, Beijing 100871, China.; ^5^ Department of Neurosurgery, Huashan Hospital Fudan University, Shanghai 200040, China.; ^6^ Department of Neurosurgery, Shanghai East Hospital, Tongji University School of Medicine, Shanghai, China.; ^7^ Department of Neurosurgery, Meizhou People’s Hospital, Meizhou Academy of Medical Sciences, Meizhou, Guangdong 514031, China.

## Abstract

**Background:** Glioblastoma, *IDH*-wild-type, is a highly invasive tumor, and prognosis largely depends on the extent of resection. Contrast-enhanced (CE) MRI often underestimates tumor borders, resulting in incomplete resection. Prospective multicenter evidence on positron emission tomography (PET)/magnetic resonance (MR)-guided resection planning in newly diagnosed glioblastoma remains limited. This study is the first nationwide, multicenter evaluation of ^18^F-fluoroethyltyrosine (FET) PET-guided glioma resection. **Methods:** We conducted a multicenter, prospective cohort study to evaluate the efficacy of a novel ^18^F-FET PET-based surgical planning system (PET/MR cross-modal tumor delineation system [PCMDS]) in glioblastoma, *IDH*-wild-type resection. The study comprised 239 patients: 115 underwent ^18^F-FET PET-guided surgery and 124 underwent CE MRI-guided surgery. The PCMDS integrated multimodal image registration and automated metabolic segmentation to delineate surgical margins. Resection outcomes were intraoperatively validated using ultrasound, MRI, and ultramicroscopic cellular imaging. **Results:** Compared with the CE MRI group, the ^18^F-FET PET group achieved a significantly higher gross total resection rate (91.3% versus 72.7%, *P* < 0.05) and improved survival outcomes (median overall survival: 19.7 versus 16.0 months, *P* = 0.0099; median progression-free survival: 12.5 versus 9.3 months, *P* = 0.0078). ^18^F-FET PET-defined margins extended beyond CE MRI-defined boundaries, and both histopathological analysis and single-cell RNA sequencing confirmed the presence of infiltrative tumor cells in ^18^F-FET PET-positive but MRI-negative regions. **Conclusion:** This multicenter study demonstrates that ^18^F-FET PET-based surgical planning significantly increases the extent of glioblastoma, *IDH*-wild-type resection and prolongs patient survival. Integrating ^18^F-FET PET into routine surgical practice could substantially improve clinical outcomes for patients with glioblastoma, *IDH*-wild-type.

## Introduction

For glioblastoma, isocitrate dehydrogenase (*IDH*)-wild-type, the current surgical paradigm emphasizes maximal safe resection—balancing radical tumor removal with functional preservation to optimize patient outcomes [1–4]. However, precise intraoperative delineation of tumor margins remains challenging because of the infiltrative nature of glioblastoma, *IDH*-wild-type, and limitations of conventional MRI in distinguishing tumor tissue from adjacent functional areas [4–8]. Conventionally, contrast-enhanced (CE) T1-weighted imaging (T1WI) is considered inadequate for delineating tumor boundaries, while T2 fluid-attenuated inversion recovery (FLAIR) tends to overestimate tumor volume [9]. ^18^F-fluoroethyltyrosine (FET) positron emission tomography (PET) has emerged as a transformative tool in glioma surgery, providing metabolic mapping that complements structural MRI [5,9–12]. By detecting up-regulated amino acid transport in tumor cells, ^18^F-FET PET improves identification of tumor boundaries and infiltrative zones [13], potentially enabling more complete resections while reducing neurological morbidity [14]. Histopathologically verified stereotactic biopsies confirmed that ^18^F-FET PET-defined tumor margins beyond CE MRI boundaries represent viable tumor tissue, demonstrating PET’s superior delineation accuracy [15]. Our clinical observations also found that patients with glioblastoma, *IDH*-wild-type, with residual postoperative FET uptake rapidly recurred at the site of uptake.

**Fig. 1.**
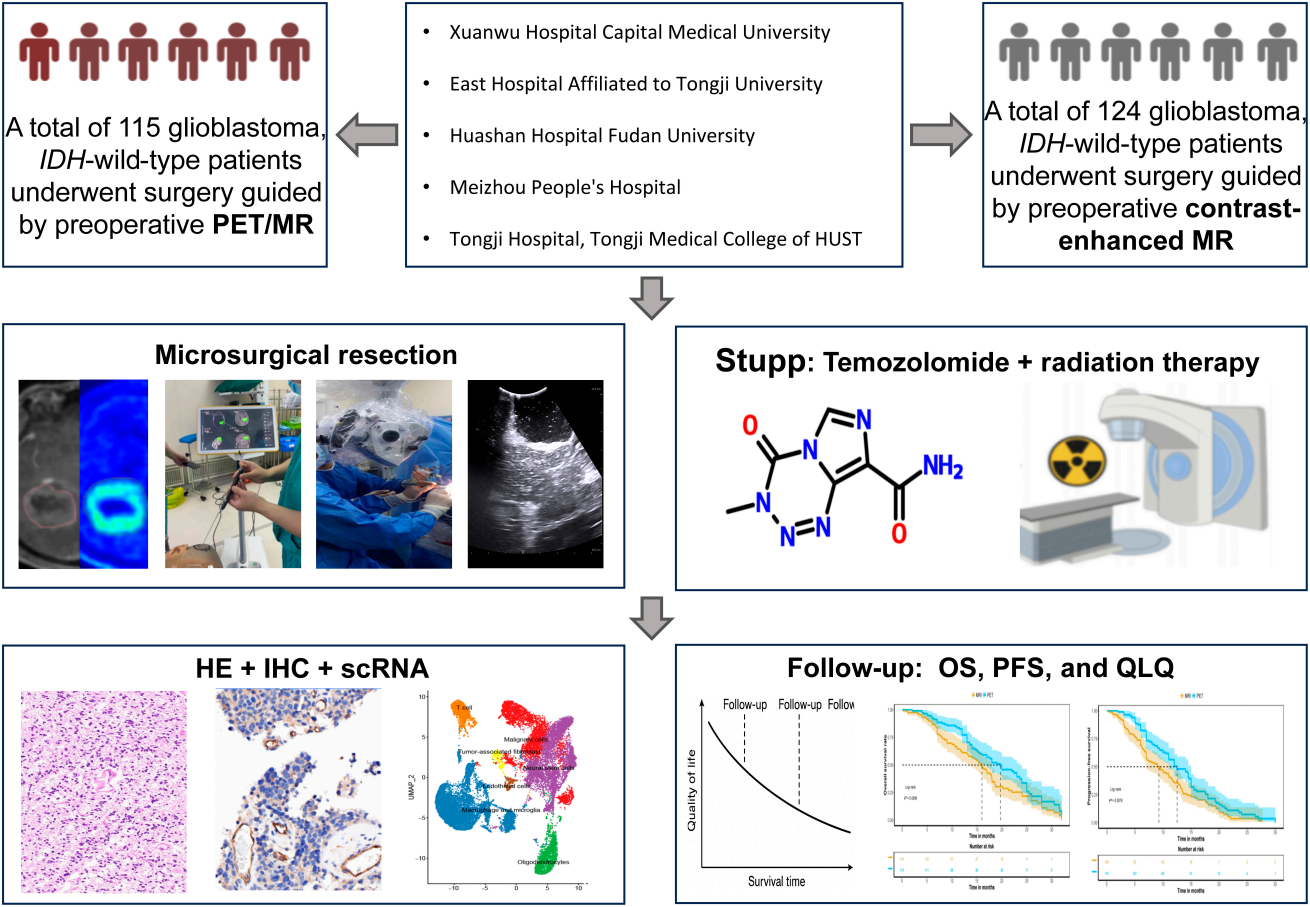
Workflow of the current study. This multicenter prospective study enrolled a total of 239 patients with glioblastoma, *IDH*-wild-type, including 124 patients who underwent surgery guided by preoperative contrast-enhanced (CE) MRI and 115 patients guided by preoperative ^18^F-fluoroethyltyrosine (FET) positron emission tomography (PET). Participating centers included Xuanwu Hospital, Capital Medical University, Huashan Hospital, Fudan University, The East Hospital Affiliated to Tongji University, Tongji Hospital, Tongji Medical College of HUST, and Meizhou People’s Hospital. All patients underwent microsurgical resection, followed by standard Stupp regimen (concurrent radiotherapy with temozolomide followed by adjuvant temozolomide) and were followed every 3 months with MRI. Follow-up protocols were harmonized across centers. Intraoperative assessments included ultrasound imaging and pathological sampling. Postoperative samples were subjected to hematoxylin and eosin (HE), immunohistochemistry (IHC), and single-cell RNA sequencing (scRNA-seq) for molecular and cellular characterization. Patients were followed up for up to 12 months with both survival (overall survival [OS] and progression-free survival [PFS]) and quality of life (QoL) assessments. QoL was measured using the European Organisation for Research and Treatment of Cancer (EORTC) QoL Questionnaire-Core 30 (QLQ-C30) and QoL Questionnaire-Brain Neoplasm Module (QLQ-BN20) at 3, 6, 9, and 12 months postoperatively. Kaplan–Meier (KM) survival curves indicated significantly better OS and PFS outcomes in the ^18^F-FET PET-guided group compared to the CE MRI-guided group. UMAP, Uniform Manifold Approximation and Projection.

**Fig. 2.**
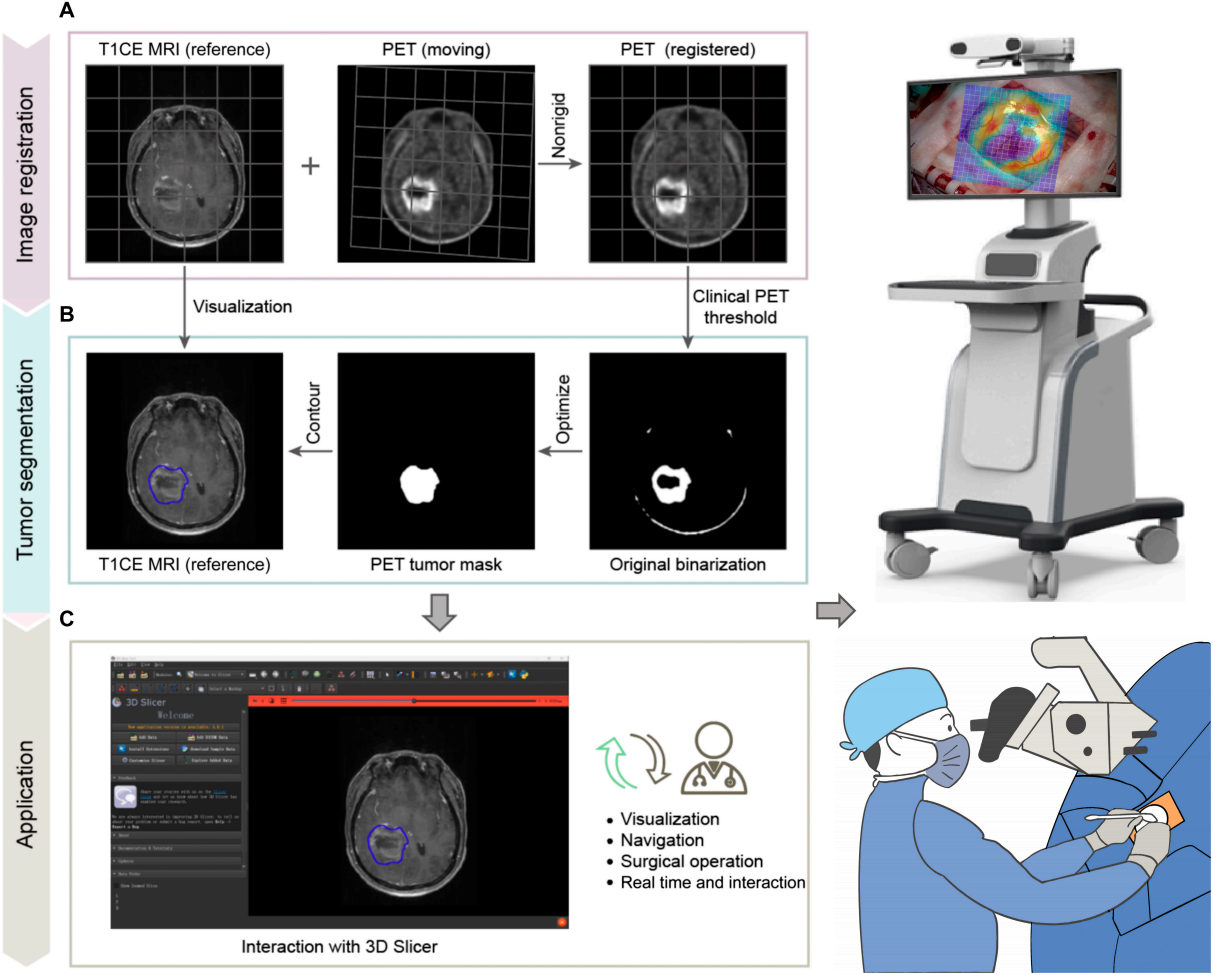
Workflow of the positron emission tomography (PET)/magnetic resonance (MR) cross-modal tumor delineation system (PCMDS). The PCMDS integrates multimodal imaging data to enhance glioblastoma boundary delineation. (A) Preprocessing of multimodal inputs including ^18^F-fluoroethyltyrosine (FET) PET and multiple MRI sequences (T1-weighted imaging [T1WI], T2WI, fluid-attenuated inversion recovery [FLAIR], and contrast-enhanced T1-weighted imaging [T1CE]) provides the structural and metabolic foundations. (B) A segmentation model receives the registered ^18^F-FET PET inputs to automatically generate tumor subregion masks, including enhancing tumor, necrotic core, peritumoral edema, and biological tumor volume. (C) The delineated tumor contours are visualized and projected intraoperatively via 3-dimensional (3D) Slicer and neuronavigation, enabling real-time interaction and surgical planning. This workflow supports high-precision tumor localization and assists neurosurgeons in performing individualized resections.

Regarding the determination of glioma boundaries using ^18^F-FET PET, there is currently much debate. In general, we define a tumor background ratio (TBR) value of 1.6 as the tumor boundary [16]. However, because ^18^F-FET PET-defined tumor margins cannot be reliably determined by visual inspection alone, we implemented a computer-assisted PET/magnetic resonance (MR) fusion-based delineation workflow integrated with surgical navigation to objectively define resection boundaries for surgery. In this workflow, the ^18^F-FET PET-defined tumor volume (*V*_PET_) was generated on registered PET images using the clinically validated TBR threshold of 1.6, followed by expert manual correction where necessary, as previously described by Song et al. [15]. Although ^18^F-FET PET is used across Western neuro-oncology practice [17], its clinical adoption in China remains limited and largely confined to small, single-center experiences. Prior domestic and international comparisons of ^18^F-FET PET-guided versus CE MRI-guided resection have typically been single-center and/or retrospective and/or underpowered, leaving a gap in high-level evidence. Prior studies have provided retrospective surgical evidence, protocol-level radiotherapy evidence, or biopsy-controlled threshold support, but prospective multicenter evidence directly evaluating a PET/MR-based surgical planning strategy in newly diagnosed glioblastoma remains limited [11,18,19]. Against this backdrop, our prospective, multicenter cohort provides the first nationwide, systematic evaluation of ^18^F-FET PET-guided resection for glioblastoma, *IDH*-wild-type, in China, generating generalizable effectiveness data and effect-size benchmarks for future randomized trials (Figs. 1 and 2). Building on European Association of Neuro-Oncology guidelines [20] and pioneering European studies [21], we investigate whether this approach enhances the extent of resection while preserving neurological function, ultimately improving survival outcomes. Our findings will provide critical evidence for incorporating metabolic imaging into China’s glioma management protocols.

## Results

### Patients and baseline characteristics

A total of 301 patients with a preoperative diagnosis of glioblastoma, *IDH*-wild-type, were prospectively enrolled, including 144 patients in the ^18^F-FET PET-guided surgical planning (^18^F-FET PET group) and 157 in the CE MRI-guided surgery (CE MRI group). In ^18^F-FET PET group, 21 patients were identified as having *IDH*-mutant or other glioma subtypes, 2 patients were lost to follow-up, 2 had incomplete imaging follow-up data, and 4 had a follow-up duration of less than 12 months. In the CE MRI group, 26 patients had *IDH*-mutant or other glioma subtypes, 1 patient was lost to follow-up, 3 had incomplete imaging follow-up data, and 3 had a follow-up duration of less than 12 months. After applying these exclusion criteria, a total of 239 patients were included in the final analysis (Fig. 1 and Tables S1 and S2). The median follow-up duration for the entire cohort was 13 months (interquartile range, 7.65 to 19.45 months). After radiological review, no obvious motion artifacts were detected in the acquired images, and image quality was considered sufficient for subsequent analysis and surgical localization. Among them, there were 115 and 124 patients in the ^18^F-FET PET and CE MRI group, respectively, representing the largest multicenter cohort to date comparing FET PET-guided versus CE MRI-guided resection in newly diagnosed glioblastoma, *IDH*-wild-type. Before weighting, baseline characteristics of patients were compared between the CE MRI and ^18^F-FET PET groups using *t* tests, nonparametric tests, and chi-square tests. No statistically significant differences were observed across age, sex, preoperative Karnofsky performance status (KPS) score, postoperative KPS score, tumor laterality, *O*-6-methylguanine-DNA methyltransferase (MGMT) promoter methylation status, telomerase reverse transcriptase (TERT) promoter mutation status, and epidermal growth factor receptor (EGFR) amplification status (*P* > 0.05; Table S2). It needs to be emphasized that the CE MRI-defined tumor volume (*V*_CE_) was comparable between the CE MRI and ^18^F-FET PET groups (39.21 [25.01 to 49.18] cm^3^ versus 39.63 [26.33 to 48.78] cm^3^, *P* = 0.847; Table S2), as was the FLAIR-defined noncontrast-enhancing abnormality volume (*V*_FLAIR_) (96.03 [72.15 to 116.53] cm^3^ versus 94.96 [78.81 to 127.50] cm^3^, *P* = 0.24; Table S2). In the ^18^F-FET PET group, the ^18^F-FET PET-defined metabolically active tumor volume (*V*_PET_) was significantly larger than the corresponding *V*_CE_ (84.65 [65.31 to 105.92] cm^3^ versus 39.21 [25.01 to 49.18] cm^3^, *P* < 0.001; Table S2). The mean Dice score between the *V*_CE_ and *V*_FET_ was 0.58 ± 0.11. TBR_max_ did not differ between the CE MRI-defined and ^18^F-FET PET-defined regions (4.14 [3.63 to 4.74] versus 4.14 [3.63 to 4.74], *P* = 1.00; Table S3) in the ^18^F-FET PET group, whereas TBR_mean_ was numerically lower in the ^18^F-FET PET-defined region than in the CE MRI-defined region, although the difference was not statistically significant (2.60 [2.23 to 3.03] versus 2.68 [2.25 to 3.11], *P* = 0.65; Table S3). In addition, all included patients had solitary lesions, and no multifocal disease was observed. Considering that this study was not a randomized controlled trial, stabilized inverse probability of treatment weighting (IPTW) [22] was further applied to reduce potential confounding. After weighting, all covariates achieved good balance, with standardized mean differences (SMDs) less than 0.1, indicating negligible residual confounding between the CE MRI and ^18^F-FET PET groups (Table S4).

### Histological and molecular validation confirms the ^18^F-FET PET metabolic boundary as the active tumor margins

**Fig. 3.**
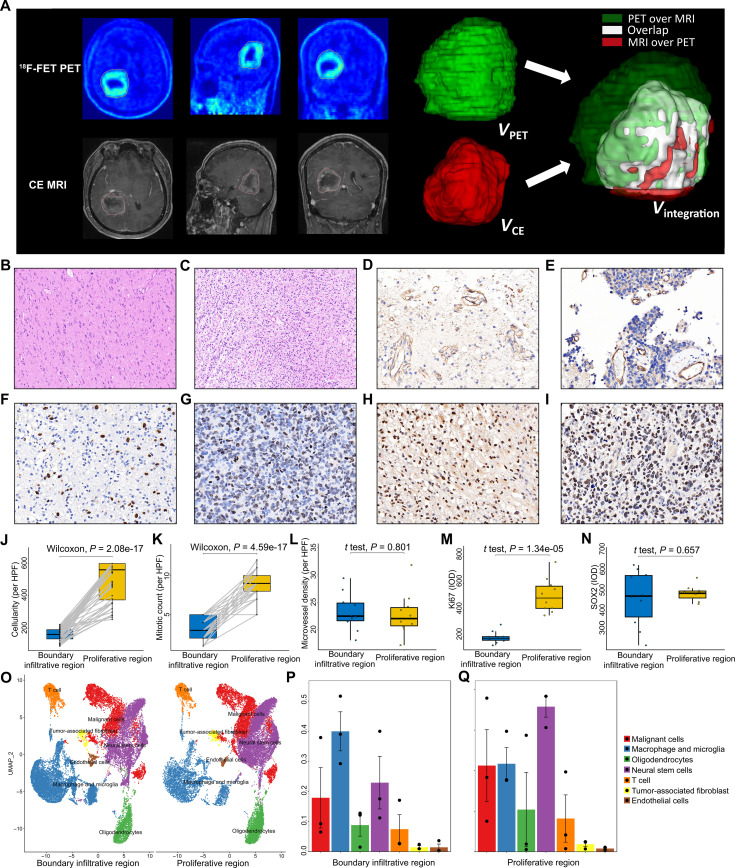
Multimodal characterization of tumor boundary heterogeneity between ^18^F-fluoroethyltyrosine (FET) positron emission tomography (PET) and contrast-enhanced (CE) MRI-defined margins. (A) In a representative glioblastoma, *IDH*-wild-type case, preoperative CE MRI and ^18^F-FET PET imaging revealed discordant tumor margins. The CE boundary (*V*_CE_, red) was narrower than the ^18^F-FET PET-defined metabolic boundary (*V*_PET_, green), indicating that CE MRI may underestimate tumor infiltration. The overlapping region between *V*_CE_ and *V*_PET_ is now displayed in gray in the integrated volume (*V*_integration_). Two concentric regions were delineated for analysis: the *V*_CE_-defined “proliferative region” and the *V*_PET_-defined “boundary-infiltrative region”. (B and C) Histopathological examination of specimens from these 2 regions revealed that the proliferative region exhibited densely packed tumor cells with necrosis and mitotic figures, while the boundary-infiltrative region displayed reduced cellularity, fewer necrotic features, and an increased presence of stromal and glial elements. (D to I) Immunohistochemistry was performed on tumor specimens from 20 representative patients to assess microvessel density (CD31), proliferative activity (Ki67), and stemness (SRY-box transcription factor 2 [SOX2]) across the 2 regions. Representative staining images are shown. (J and K) Quantitative histopathological analysis showing significantly higher cellularity per high-power field (HPF) (J) and mitotic count per 10 HPFs (K) in the proliferative region than in the boundary-infiltrative region. (L) No significant differences were observed in CD31-positive microvessel density between the 2 regions (*P* > 0.05). (M) Quantitative analysis (per high-power field [HPF]) demonstrated that the Ki67 immunoreactive optical density (IOD) was significantly higher in the proliferative region compared to the boundary-infiltrative region (*P* < 0.05), suggesting increased tumor cell proliferation at the contrast-enhancing margin. (N) No significant differences were observed in SOX2 IOD between the 2 regions (*P* > 0.05). (O to Q) Single-cell RNA sequencing (scRNA-seq) of paired tissue samples from 3 patients with glioblastoma, *IDH*-wild-type, revealed distinct cellular compositions between the 2 compartments. The proliferative region was enriched in malignant glioma cells, while the boundary-infiltrative region contained a higher proportion of macrophages and T cells, highlighting microenvironmental divergence at the tumor edge.

To validate of the active tumor margins in glioblastoma, *IDH*-wild-type, we integrated multimodal imaging, histopathology, single-cell RNA sequencing (scRNA-seq), and immunohistochemistry (IHC) across different peritumoral regions defined by CE MRI and ^18^F-FET PET—an approach that, to our knowledge, has not been previously reported in the context of PET-guided glioma surgery. In a representative glioblastoma, *IDH*-wild-type case, preoperative CE MRI and ^18^F-FET PET imaging revealed discordant tumor boundaries (Fig. 3A). The CE margin (*V*_CE_) was notably narrower than the ^18^F-FET PET-derived metabolic boundary (*V*_PET_), suggesting that CE MRI may underestimate the true extent of tumor boundary. This spatial discrepancy informed the designation of 2 concentric regions for further analysis: the CE tumor rim (*V*_CE_, referred to as the “proliferative region”) and the outer metabolic zone (*V*_PET_, referred to as the “boundary-infiltrative region”).

Histological examination of tissue from these regions provided further confirmation (Fig. 3B and C). The proliferative region contained densely packed tumor cells with areas of necrosis and increased mitotic activity, whereas the boundary-infiltrative region showed reduced cellularity, fewer necrotic features, and a more prominent presence of stromal and glial elements (Fig. 3J and K). The PET-defined margin represents the active tumor margin—a fact underscored by the architectural heterogeneity observed between it and the CE MRI border.

To further characterize the tumor microenvironment across spatial compartments, we performed immunohistochemical staining on surgically resected specimens from the proliferative (10 cases) and boundary-infiltrative regions (10 cases) in 20 representative glioblastoma, *IDH*-wild-type cases. CD31 was used to assess microvessel density (Fig. 3D and E), Ki67 to evaluate proliferative activity (Fig. 3F and G), and SRY-box transcription factor 2 (SOX2) to indicate stemness (Fig. 3H and I). Quantitative histopathological analysis showed significantly higher cellularity per HPF and mitotic count per 10 HPFs in the proliferative region than in the boundary-infiltrative region (Fig. 3J and K). No significant differences were observed in CD31-positive microvessel density between the 2 regions (Fig. 3L, *P* > 0.05). Quantitative analysis revealed that the proliferative region exhibited significantly higher Ki67 immunoreactive optical density (IOD) values compared to the boundary-infiltrative region (Fig. 3M, *P* < 0.05), indicating enhanced tumor cell proliferation. No significant differences were observed between the 2 regions in terms of SOX2 IOD values (Fig. 3N, *P* > 0.05). Collectively, these multimodal results confirm that the ^18^F-FET PET-defined tumor boundary encompasses a distinct infiltrative microenvironment beyond the CE rim, reinforcing the value of ^18^F-FET PET in surgical planning and biological interpretation of glioma margins.

To dissect the cellular architecture at scRNA-seq resolution, paired samples from the proliferative and infiltrative regions in 3 patients with glioblastoma, *IDH*-wild-type, underwent scRNA-seq profiling (Fig. 3O to Q). Although both regions shared major cell populations—such as oligodendrocyte lineage cells, astrocytes, and immune cells—the proliferative region exhibited a higher proportion of malignant glioma cells, while the boundary-infiltrative region was modestly enriched for macrophages and T cells, highlighting microenvironmental divergence across spatial compartments.

### Real-time intraoperative imaging techniques validates the accuracy of tumor resection guided by ^18^F-FET PET

To further evaluate the surgical validity of tumor boundaries defined by CE MRI versus ^18^F-FET PET, we initially employed 3 real-time intraoperative techniques—ultrasound, ultramicroscopic cellular imaging, and MRI—to assess the extent of resection in separate patient cohorts. Gross total resection (GTR) was defined as the absence of any detectable residual tumor on the corresponding images obtained from intraoperative ultrasound, ultramicroscopic cellular imaging, and MRI.

**Fig. 4.**
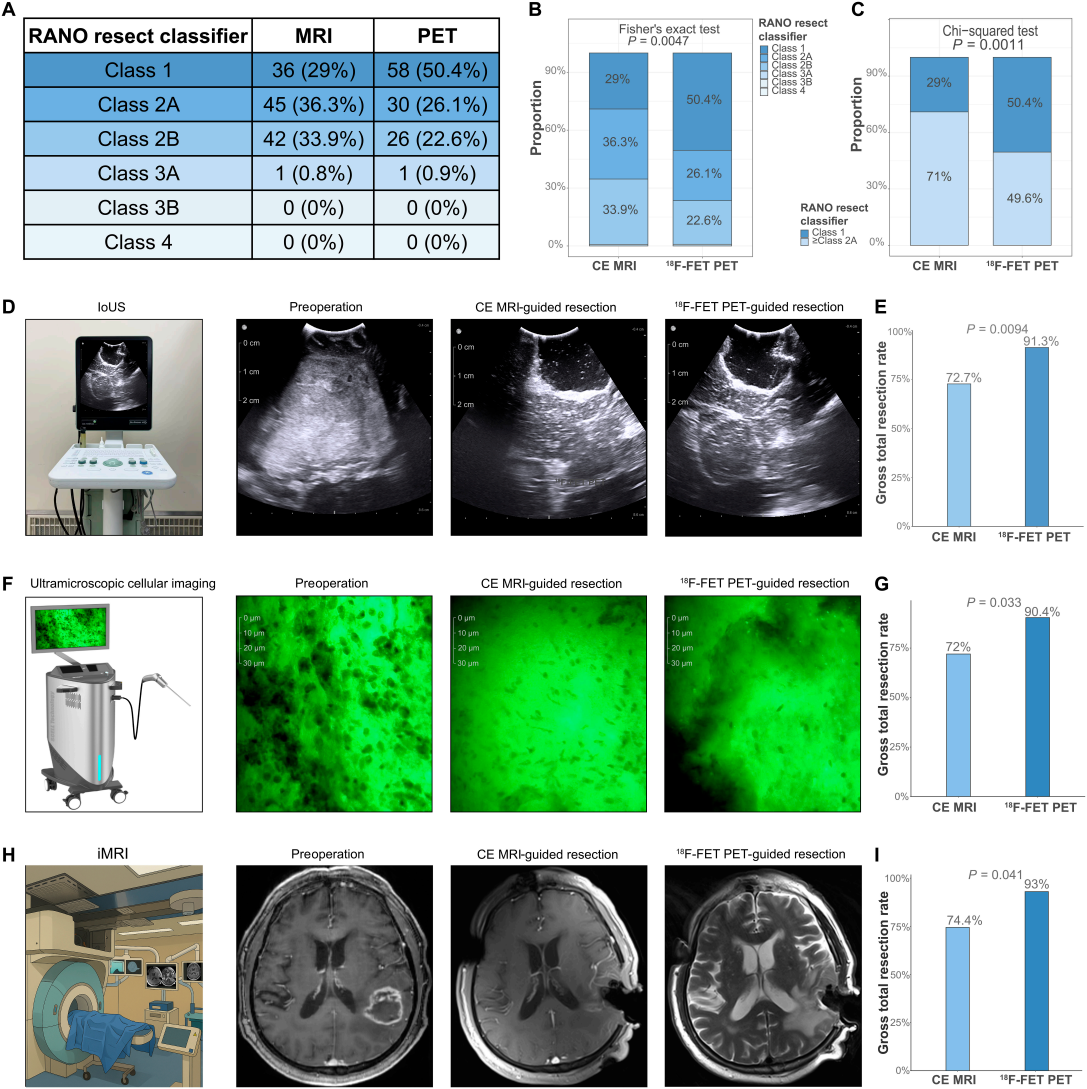
Extent-of-resection assessment and real-time intraoperative validation of tumor resection guided by ^18^F-fluoroethyltyrosine (FET) positron emission tomography (PET). (A) Distribution of Response Assessment in Neuro-Oncology (RANO) resect classifier categories in the CE MRI-guided and ^18^F-FET PET-guided resection groups. Compared with the contrast-enhanced (CE) MRI group, the ^18^F-FET PET group showed a higher proportion of Class 1 resections and lower proportions of Class 2A and Class 2B resections. (B) Stacked bar plot showing the proportional distribution of all RANO resect classifier categories between the two groups. The overall distribution differed significantly between the CE MRI and ^18^F-FET PET groups, as assessed by Fisher’s exact test. (C) Dichotomized comparison of optimal resection status, defined as Class 1 versus Class ≥ 2A. The ^18^F-FET PET group achieved a higher proportion of Class 1 resections than the CE MRI group. (D and E) Intraoperative ultrasound. Intraoperative ultrasound (IoUS) was used intraoperatively in 135 patients (CE MRI group, *n* = 66; ^18^F-FET PET group, *n* = 69) to assess residual disease. (D) IoUS console and a representative sequence from the same case: preoperation (baseline), after CE MRI-guided resection (persistent hyperechoic tissue compatible with residual tumor), and after ^18^F-FET PET-guided resection (no hyperechoic remnant, consistent with gross total resection). (E) Group outcomes: Gross total resection (GTR) by IoUS was 72.7% (48 of 66) for CE MRI versus 91.3% (63 of 69) for ^18^F-FET PET (P = 0.0094). (F and G) Ultramicroscopic cellular imaging. High resolution ultramicroscopic cellular imaging was performed in 102 patients (CE MRI group, *n* = 50; ^18^F-FET PET group, *n* = 52) to detect residual tumor cells at the cavity edge. (F) The device and a representative sequence: preoperation, after CE MRI-guided resection (persistence of tumor cells indicating incomplete resection), and after ^18^F-FET PET-guided resection (no residual tumor cells, consistent with complete excision). (G) Group-level results: cell-free margins in 72.0% (36/50) of CE MRI cases versus 90.4% (47 of 52) of ^18^F-FET PET cases (*P* = 0.033). (H and I) Intraoperative MRI. Intraoperative MRI (iMRI) was performed in 86 patients (CE MRI group, *n* = 43; ^18^F-FET PET group, *n* = 43). (H) iMRI suite and a representative sequence: preoperation CE T1 image, after CE MRI-guided resection (residual nodular enhancement), and after extension to the ^18^F-FET PET-defined margin (no residual enhancement). (I) GTR rates confirmed by iMRI: 74.4% (32 of 43) for CE MRI versus 93.0% (40 of 43) for ^18^F-FET PET (*P* = 0.041).

Intraoperative ultrasound (IoUS) was applied in 135 patients (CE MRI group, *n* = 66; ^18^F-FET PET group, *n* = 69) to dynamically visualize residual tumor tissue during surgery. Representative images from a patient with glioblastoma, *IDH*-wild-type, are shown in Fig. 4D. The baseline preresection IoUS (preoperation) is followed by IoUS immediately after resection guided by CE MRI-defined margins, which demonstrates persistent hyperechoic tissue along the cavity compatible with residual tumor (incomplete resection). A subsequent IoUS scan obtained after resection extended to the ^18^F-FET PET-defined margins shows no residual hyperechoic signal, consistent with GTR. Group-level outcomes are summarized in Fig. 4E: The GTR rate was 91.3% (63 of 69) in the ^18^F-FET PET group versus 72.7% (48 of 66) in the CE-MRI group (*P* = 0.0094).

Ultramicroscopic cellular imaging, a high-resolution transmission electron microscopy technique, was applied to 102 patients (CE MRI group, *n* = 50; ^18^F-FET PET group, *n* = 52) to evaluate the presence of residual tumor cells at the resection margins. In a representative case (Fig. 4F), the device is shown at left, followed by sequential images from the same site: preoperation (baseline cellularity), after CE MRI-guided resection (persistence of tumor cells consistent with incomplete resection), and after ^18^F-FET PET-guided resection (no visible residual tumor cells, consistent with gross total excision). Cohort-level results are summarized in Fig. 4G: Absence of residual cells was achieved in 47 of 52 (90.4%) patients in the ^18^F-FET PET group versus 36 of 50 (72.0%) in the CE MRI group (*P* = 0.033).

Intraoperative MRI (iMRI) was performed in a total of 86 patients (CE MRI group, *n* = 43; ^18^F-FET PET group, *n* = 43). Figure 4H depicts the iMRI suite and a representative case in sequence: the preresection CE T1 image (preoperation), the first postresection scan following CE MRI-guided resection demonstrating persistent nodular enhancement compatible with residual tumor, and a subsequent scan obtained after extending resection to the ^18^F-FET PET-defined margins showing no residual enhancement, consistent with GTR. Cohort-level outcomes are summarized in Fig. 4I: GTR was achieved in 40 of 43 (93%) patients in the ^18^F-FET PET group versus 32 of 43 (74.4%) in the CE MRI group (*P* = 0.041).

Together, these intraoperative evaluations demonstrate that ^18^F-FET PET-defined tumor boundaries are more reliable in achieving complete resection of glioblastoma, *IDH*-wild-type, across 3 distinct intraoperative validation techniques. The consistency of superior GTR rates in the ^18^F-FET PET group across all 3 methods highlights the biological and clinical relevance of ^18^F-FET PET-guided surgical planning.

### Response assessment in neuro-oncology resect classifier analysis

When reevaluated according to the response assessment in neuro-oncology (RANO) resect classifier [23], the ^18^F-FET PET group showed a more favorable extent-of-resection distribution than the CE MRI group. In the CE MRI group (*n* = 124), 36 patients (29.0%) were classified as class 1, 45 (36.3%) as class 2A, 42 (33.9%) as class 2B, and 1 (0.8%) as class 3A; no class 3B or class 4 cases were identified. In the ^18^F-FET PET group (*n* = 115), 58 patients (50.4%) were classified as class 1, 30 (26.1%) as class 2A, 26 (22.6%) as class 2B, and 1 (0.9%) as class 3A, with no class 3B or class 4 cases. The overall distribution of RANO resect categories differed significantly between groups (Fig. 4A and B, Fisher’s exact test, *P* = 0.0047). In a simplified sensitivity analysis comparing class 1 versus ≥class 2A, the PET group still showed a significantly higher proportion of class 1 resections (Fig. 4C, 50.4% versus 29.0%, chi-square test, *P* = 0.0011).

### ^18^F-FET PET-guided tumor resection improves survival outcomes in patients with glioblastoma, *IDH*-wild-type

**Fig. 5.**
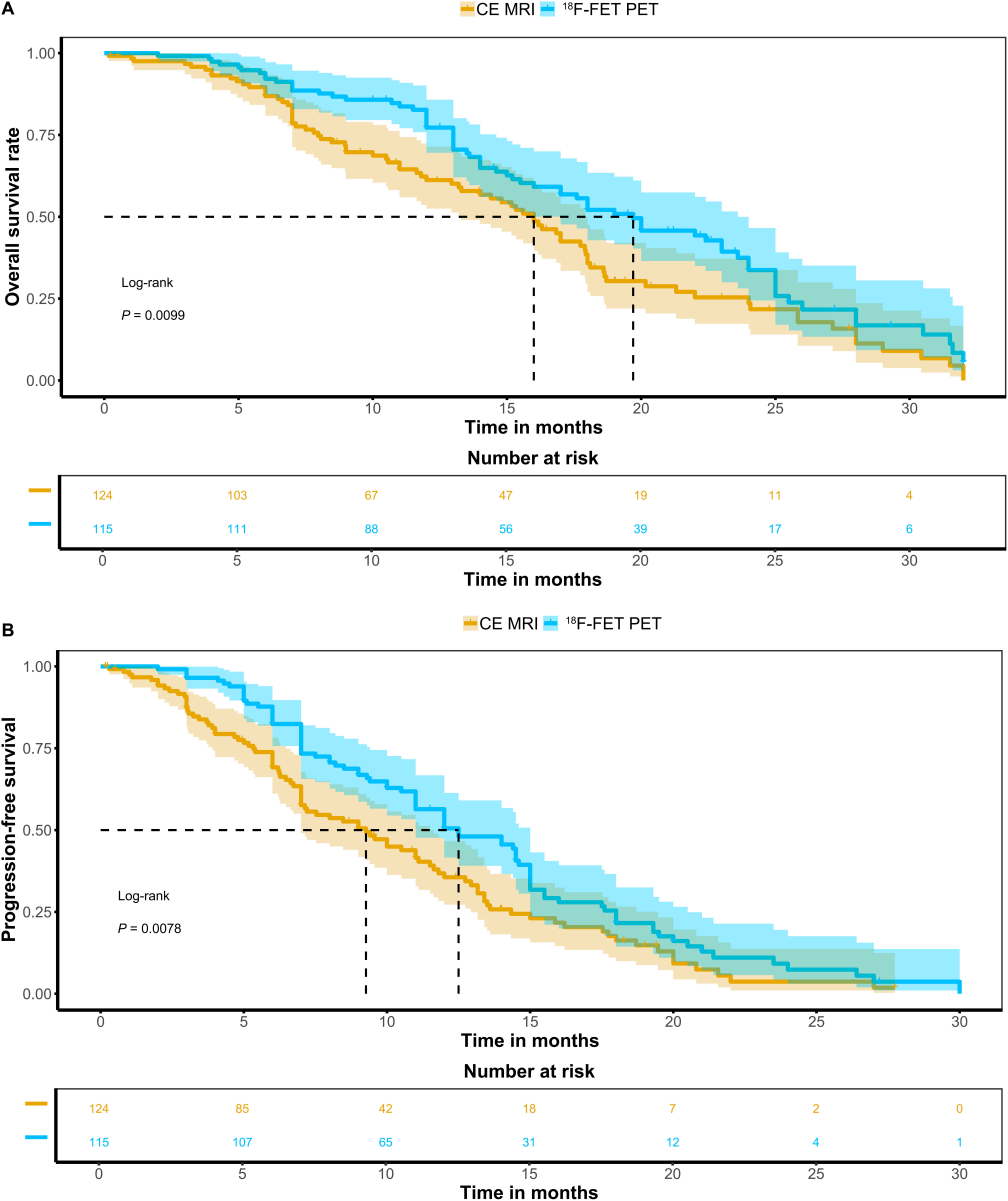
Kaplan–Meier (KM) survival analysis comparing the ^18^F-fluoroethyltyrosine (FET) positron emission tomography (PET) and contrast-enhanced (CE) MRI groups. Shaded areas represent 95% confidence intervals (CIs). Numbers at risk at each time point are listed below the x-axis. (A) KM curve for overall survival (OS). The ^18^F-FET PET group showed a significantly longer median OS of 19.7 months compared to 16.0 months in the MRI group (*P* = 0.0099). (B) KM curve for progression-free survival (PFS). The ^18^F-FET PET group had a significantly longer median PFS of 12.5 months, whereas the CE MRI group had a median PFS of 9.3 months (*P* = 0.0078).

The patients in the ^18^F-FET PET group demonstrated significantly improved overall survival (OS) and progression-free survival (PFS) compared to the CE MRI group. The median OS was 19.7 months in the ^18^F-FET PET group versus 16.0 months in the CE MRI group (*P* = 0.0099), while the median PFS was 12.5 months versus 9.3 months, respectively (*P* = 0.0078). Kaplan–Meier (KM) survival curves (Fig. 5A and B) demonstrated a consistently superior survival trend in the ^18^F-FET PET group throughout the follow-up period. Similar findings were observed following IPTW adjustment: The IPTW-adjusted median OS was 19.7 months in the ^18^F-FET PET group and 16.0 months in the CE MRI group (*P* = 0.01); the IPTW-adjusted median PFS was 12.5 months versus 9.3 months, respectively (*P* = 0.01). The IPTW-adjusted KM survival curves are presented in Fig. S1.

### No difference in quality of life between CE MRI and ^18^F-FET PET groups

Quality of life (QoL) outcomes were assessed using the European Organisation for Research and Treatment of Cancer (EORTC) QoL Questionnaire-Core 30 (QLQ-C30) and QoL Questionnaire-Brain Neoplasm Module (QLQ-BN20). Before weighting, comparisons of CE MRI and ^18^F-FET PET groups at 3, 6, 9, and 12 months postoperatively were performed using *t* tests or Wilcoxon rank sum tests, and all *P* values were greater than 0.05 (Tables S5 and S6) except for constipation at 6 months. After IPTW, weighted comparisons were conducted within the survey design framework, with adjusted means and 95% confidence intervals (CIs) estimated for each group. Between-group differences were derived from weighted regression models, and all *P* values remained greater than 0.05 (Table S7 and S8). These findings indicate that there were no significant differences in QoL scores between the CE MRI and ^18^F-FET PET groups, regardless of weighting.

## Discussion

Our findings provide the first prospective multicenter evidence supporting the clinical relevance of ^18^F-FET PET-guided resection planning in newly diagnosed glioblastoma, *IDH*-wild-type, with innovative multidimensional validation including histopathology, IHC, scRNA-seq, and 3 real-time intraoperative techniques. This first multicenter study demonstrates that ^18^F-FET PET-guided surgery using the PET/MR cross-modal tumor delineation system (PCMDS) represents an important advancement in the management of glioblastoma, *IDH*-wild-type, with superior surgical outcomes and survival benefits compared to CE MRI-guided approaches, while maintaining comparable QoL metrics. These results have important implications for clinical practice and future research directions in surgical neuro-oncology.

The key strength of this study lies in its comprehensive validation approach. By using 3 independent real-time intraoperative techniques (ultrasound, ultramicroscopic cellular imaging, and MRI), we established the reliability of ^18^F-FET PET-defined tumor boundaries with unprecedented rigor [13,24]. The consistently higher GTR rates achieved in the ^18^F-FET PET group (91.3% versus 72.7% by IoUS) across all validation modalities strongly supports the clinical utility of metabolic imaging for surgical planning [14]. This finding is particularly important given the well-established correlation between extent of resection and survival outcomes in glioblastoma, *IDH*-wild-type [25,26]. Our biological analyses not only validated the tumor activity boundaries defined by ^18^F-FET PET but also revealed critical molecular features of the tumor microenvironment’s heterogeneity. The scRNA-seq data demonstrated distinct cellular compositions between CE MRI and ^18^F-FET PET-defined margins, with the former capturing infiltrative tumor cells that would otherwise be missed [27,28]. These findings align with emerging understanding of glioblastoma, *IDH*-wild-type biology, where tumor cells frequently extend beyond CE regions [29] and which implies that PET-defined regions necessitate aggressive therapeutic approaches, such as surgical resection. The ultrastructural evidence from electron microscopy further corroborated these observations, showing residual tumor cells at CE MRI-defined margins that were completely resected using ^18^F-FET PET guidance [30]. The RANO resect analysis further supports the surgical relevance of PET-guided boundary definition. Given that postoperative residual tumor burden and RANO resect categories are strongly associated with prognosis in glioblastoma, *IDH*-wild-type [23], the higher proportion of class 1 resections in the PET group suggests that metabolically guided resection may facilitate a more complete tumor removal within the RANO framework. Importantly, the difference between groups was driven mainly by a shift from class 2A/2B toward class 1, rather than by an excess of poor resection categories, as no class 3B or class 4 cases were observed in either group.

The survival outcomes in our study represent a meaningful clinical advance. The 3.7-month improvement in median OS (19.7 months versus 16.0 months) and 3.2-month advantage in PFS (12.5 months versus 9.3 months) compare favorably with recent advances in glioblastoma, *IDH*-wild-type treatment [20,31]. Importantly, these benefits were achieved without compromising neurological function or QoL, as evidenced by comparable QoL scores between groups [32]. This suggests that metabolic boundary delineation enables more aggressive resection while preserving eloquent brain areas—a crucial balance in neuro-oncological surgery [33]. The development of the PCMDS system addresses several longstanding challenges in glioma surgery. By integrating automated segmentation with deformable registration algorithms, our platform overcomes key limitations of visual PET interpretation [21]. The implementation within 3-dimensional (3D) Slicer ensures clinical practicality, allowing real-time adjustments during surgery [34]. This technological innovation represents a major step toward precision neurosurgery, where molecular imaging complements traditional anatomical guidance [35].

The volumetric and semiquantitative analyses further clarify the relationship between CE MRI-defined tumor compartments and FET-defined metabolically active tumor tissue. In the ^18^F-FET PET group, TBR_max_ was identical between the CE MRI-defined region and the PET-defined active volume, and TBR_mean_ was also comparable, with no statistically detectable difference between regions. These findings suggest that ^18^F-FET PET uptake was not clearly higher in the CE MRI-defined proliferative core than in the ^18^F-FET PET -defined metabolically active volume. This observation is biologically plausible because FET-positive tissue beyond the contrast-enhancing margin may still contain metabolically active infiltrative glioma rather than representing merely low-uptake peripheral tissue. This interpretation is consistent with current joint European Association of Nuclear Medicine/European Association of Neuro-Oncology/RANO/Society of Nuclear Medicine and Molecular Imaging recommendations, which emphasize that amino acid PET complements MRI by delineating biologically active tumor beyond conventional contrast enhancement, as well as biopsy-controlled studies showing that ^18^F-FET PET-positive regions outside CE MRI may correspond to infiltrative tumor tissue [11,15,17].

At the same time, our data suggest that the observed survival difference between groups is unlikely to be explained simply by an imbalance in baseline CE MRI-defined tumor burden. The 2 groups had comparable baseline *V*_CE_ and *V*_FLAIR_, and both variables remained well balanced after IPTW adjustment, indicating similar CE MRI-based preoperative tumor load between treatment groups. In addition, although the ^18^F-FET PET-defined active volume was larger than *V*_CE_ in the great majority of patients, this was not universal: In our cohort, only a minority of patients showed larger CE MRI-defined volumes than ^18^F-FET PET-defined active volumes. This finding is in line with previous reports showing that ^18^F-FET PET-defined tumor volume is frequently, but not invariably, larger than contrast enhancement. Taken together, these results support the view that CE MRI and ^18^F-FET PET describe overlapping but nonidentical tumor compartments and that ^18^F-FET PET provides clinically relevant information particularly at the infiltrative tumor boundary [15,17,36].

Several limitations should be acknowledged. First, although IPTW was applied to adjust for measured baseline imbalances, the nonrandomized design remains inherently susceptible to residual confounding and selection bias. In particular, because of the success rate of ^18^F-FET tracer production, logistical constraints, and the rapid clinical progression of glioblastoma, *IDH*-wild-type, not all potentially eligible patients were able to undergo preoperative ^18^F-FET PET imaging. Consequently, the ^18^F-FET PET and CE MRI groups did not arise from fully symmetric enrollment pathways, and the CE MRI group was not enrolled under the same registered parent protocol as the ^18^F-FET PET cohort. Although key measured clinical and molecular covariates, including age, preoperative KPS, tumor size, MGMT promoter methylation, TERT promoter mutation, and EGFR amplification status, were incorporated into the IPTW model, unmeasured confounding cannot be completely excluded. While this approach enhances internal validity, formal stratified or interaction subgroup analyses were not performed. Future studies with larger sample sizes may benefit from such analyses to explore heterogeneous treatment effects. Second, as a prospective multicenter study, potential intercenter heterogeneity remains inevitable. Variations in imaging equipment, acquisition protocols, institutional experience, and patient case mix may have introduced variability in outcomes. Case accrual was not evenly distributed across centers, with a relatively larger contribution from the leading referral center, which may increase the possibility that some findings were influenced by center-level effects. Collectively, the available analytical results, including the IPTW-based adjustment, supported the overall robustness of the findings despite the uneven distribution of case accrual across centers. In our next prospective study, we will further extend the follow-up duration and make additional efforts to optimize multicenter enrolment balance and prespecified center-stratified analyses. While the multicenter design improves generalizability, it may also confound certain outcome assessments. Future studies should also implement fully harmonized imaging protocols, centralized data curation, and core-lab-based quality control to improve reproducibility and consistency across sites. Third, although the cohort met the predefined follow-up requirement and the median follow-up duration was 13 months (interquartile range, 7.65 to 19.45 months), longer follow-up will still be valuable to confirm the durability of the observed OS benefit. Fourth, the requirement for specialized PET/MR infrastructure may limit immediate widespread adoption, particularly in resource-limited settings [6]. Fifth, postoperative ^18^F-FET PET was not routinely performed. Although postoperative MRI allowed assessment of incomplete resection, residual metabolically active tumor could not be systematically evaluated after surgery. In a small exploratory subset of patients with MRI-defined incomplete resection (RANO 2B + 3A), postoperative PET was available in only 13 of 43 cases, which was insufficient for a reliable prognostic subgroup analysis. Future prospective studies with routine postoperative ^18^F-FET PET are needed to determine whether metabolically active residual disease provides additional prognostic information beyond MRI-defined residual tumor. In addition, the study focused on newly diagnosed glioblastoma, *IDH*-wild-type, and further research is needed to evaluate this approach in recurrent disease or lower-grade gliomas [17]. Future directions should focus on several key areas: (a) cost-effectiveness analyses to guide healthcare resource allocation [37]; (b) integration with emerging technologies such as artificial intelligence for automated boundary detection; (c) combination with novel therapeutic agents targeting the infiltrative tumor compartment [38]; and (d) multicenter randomized trials to establish definitive evidence of superiority [39].

In conclusion, this study establishes ^18^F-FET PET-guided surgery as a new standard of care for glioblastoma, *IDH*-wild-type resection. The PCMDS system provides neurosurgeons with a powerful tool to achieve more complete tumor removal while minimizing functional deficits. These findings underscore the importance of metabolic imaging in modern neuro-oncology and pave the way for further innovations in precision brain tumor surgery.

## Methods

### Study design and participants

This study was a prospective, nonrandomized, matched controlled multicenter cohort study conducted at 5 institutions equipped with ^18^F-FET PET between 2023 February 1 and 2025 September 30. Patients diagnosed with newly diagnosed glioblastoma, *IDH*-wild-type, were prospectively enrolled and assigned to 1 of 2 groups based on the preoperative imaging strategy used for surgical planning: the ^18^F-FET PET and the CE MRI groups. All patients in the ^18^F-FET PET group underwent ^18^F-FET PET imaging preoperatively, while patients in the CE MRI group received CE MRI only. Notably, patients in the ^18^F-FET PET group were derived from a large prospective registry-based cohort, which had received ethics approval and was registered at ClinicalTrials.gov (identifier: NCT06092125). All patients received the standard Stupp regimen (concurrent radiotherapy with temozolomide followed by adjuvant temozolomide) and were followed up every 3 months with MRI. Follow-up protocols were harmonized across centers.

All eligible patients were consecutively enrolled according to predefined inclusion and exclusion criteria. The date of surgery was defined as the enrollment date (time zero). This study used a prospective contemporaneous matching strategy: Whenever a patient undergoing PET-guided preoperative surgical planning was enrolled into the ^18^F-FET PET group, one control patient undergoing conventional MR-guided surgical planning was selected from the same contemporaneous period for the CE MRI group. Thus, matching was performed at the time each ^18^F-FET PET case entered the study rather than after accrual of the entire cohort. Enrollment timing between the ^18^F-FET PET and the CE MRI groups was kept as closely synchronized as possible to minimize potential bias related to calendar time, perioperative workflow changes, and center-specific practice variations. All matches were established according to prespecified rules, and once a matched pair was confirmed, the pairing was locked and not subsequently altered or reassigned.

Matching was performed within the same study center and within the same calendar-time window. Specifically, eligible CE MRI controls were required to have undergone surgery on the same day as, or within 5 d after, the corresponding ^18^F-FET PET case. Matching was based on predefined hard screening criteria, with no restriction on the matching ratio. Candidate CE MRI controls were required to have the same sex as the ^18^F-FET PET case (male-to-male and female-to-female exact matching), the same tumor laterality (left or right), an age difference of no more than 5 years, a preoperative KPS difference of no more than 10 points, and a difference in preoperative maximal tumor diameter of no more than 1 cm. All CE MRI patients meeting all of these criteria for a given ^18^F-FET PET case were included as matched controls. If more than one CE MRI candidate satisfied the predefined matching criteria, all eligible candidates were included. If no fully matched CE MRI control could be identified within the same center and within 5 d after the ^18^F-FET PET case, the ^18^F-FET PET case remained in the study cohort, whereas no CE MRI control was included for that case.

All eligible patients were included in the analytical framework, and IPTW was applied to adjust for baseline imbalances and reduce confounding associated with the nonrandomized design. The propensity score was estimated using a logistic regression model that included the following baseline covariates: age, sex, tumor size, tumor laterality (left versus right), preoperative and postoperative KPS, Ki67 index, MGMT promoter methylation status, TERT promoter mutation status, and EGFR amplification status. Regardless of whether significant differences existed between the 2 groups for the above covariates, IPTW adjustment was performed to further minimize potential confounding and ensure comparability. For continuous variables such as age, the Shapiro–Wilk test was used to assess normality. As the original distribution of age did not meet the assumption of normality, nonparametric tests (e.g., Mann–Whitney *U* test) were used for unweighted comparisons. After IPTW adjustment, weighted *t* tests were used for group comparisons of continuous variables, as the weighting process tends to approximate a pseudo-population with improved distributional properties.

## Ethical Approval

This study was approved by the Ethics Committee of Xuanwu Hospital, Capital Medical University (approval no. [2023]044), and written informed consent was obtained from all participants.

### Sample size calculation

The sample size was estimated on the basis of survival outcomes from a preliminary retrospective cohort, which included 34 patients in the CE MRI group and 32 patients in the ^18^F-FET PET group, with a minimum follow-up of 12 months. In this pilot dataset, the ^18^F-FET PET group showed a reduced risk of death compared with the CE MRI group, with a hazard ratio of 0.54 (95% CI, 0.29 to 1.00; *P* = 0.046). The median survival times were 16 months for the CE MRI group and 19 months for the ^18^F-FET PET group. Using these parameters, sample size estimation was performed with PASS software (version: 15.0), assuming a 2-sided *α* of 0.05, 85% power, a 1:1 allocation ratio, a planned accrual period of 20 months, and an additional follow-up period of at least 12 months for the last enrolled patient, resulting in a total study duration of 32 months (2023 February 1 to 2025 September 30). After allowing for an anticipated dropout rate of 10%, the final target sample size was 211 patients (^18^F-FET PET group = 106, CE MRI group = 105).

Sample allocation across centers was determined in proportion to their historical patient volume. Given that center 1 was expected to contribute approximately 10 times as many eligible patients as each of the other 4 centers, the planned allocation ratio was approximately 10:1:1:1:1. Accordingly, center 1 was expected to enroll approximately 151 to 171 patients, whereas each of the remaining 4 centers was expected to enroll approximately 10 to 15 patients. Minor adjustments were allowed during the actual enrollment process according to screening, matching feasibility, and follow-up status.

### Eligibility criteria

Inclusion criteria were age of ≥18 and ≤60 years; supratentorial lesion suspected to be glioblastoma, *IDH*-wild-type, on imaging; histopathologically confirmed glioblastoma, *IDH*-wild-type, based on the World Health Organization Classification of Tumours of the Central Nervous System (2021, 5th edition); KPS of ≥70; no prior treatment for glioma; and no history of other malignancies. Exclusion criteria included *IDH*-mutant or other glioma subtypes; multifocal or infratentorial tumors; incomplete clinical or imaging data; and lost to follow-up. In addition, nonenhancing glioblastomas were excluded from the present cohort.

### Primary and secondary end points

The primary end points of this study were OS and PFS. The secondary end point was postoperative QoL, assessed using the EORTC QLQ-C30 and QLQ-BN20 at predefined time points [40–43].

The OS and PFS were analyzed using the KM method, and group differences were assessed with the log-rank test. Hazard ratios and 95% CIs were estimated using Cox proportional hazards regression models. IPTW was incorporated into the Cox models to generate adjusted estimates of survival benefit. All statistical analyses were conducted using R (version 4.1.3) with the survey and survival packages.

The QoL outcomes were assessed using the EORTC QLQ-C30 and QLQ-BN20 at 3, 6, 9, and 12 months after surgery. Before weighting, comparisons of CE MRI and ^18^F-FET PET groups at 3, 6, 9, and 12 months postoperatively were performed using *t* tests or Wilcoxon rank sum tests. After IPTW, weighted comparisons were conducted within the survey design framework, with adjusted means and 95% CIs estimated for each group. Between-group differences were derived from weighted regression models.

### Hybrid PET/MR scanning and image processing

^18^F-FET PET was produced and applied as described previously [15,44,45]. For patients undergoing ^18^F-FET PET and CE MRI, images were acquired on 3-T hybrid PET/MR systems, including GE Signa (GE Healthcare, USA) and uPMR 790 (United Imaging, China), as detailed in Supplementary Methods. A standard MRI protocol comprising 3D T2 FLAIR (repetition time/echo time = 9,000/maximum), pre- and postgadolinium 3D T1WI (8.5/3.2) and axial T1WI, T2WI, FLAIR, and diffusion-weighted imaging was used.

### ^18^F-FET PET cross-modal tumor delineation system

We developed a Python-based intelligent fusion system that integrates CE MRI and ^18^F-FET PET imaging to leverage the high spatial resolution of CE MRI alongside the superior tumor delineation capabilities of ^18^F-FET PET (Fig. 1). The proposed system consists of 3 key components: (a) multimodal image registration, (b) automated tumor segmentation, and (c) a clinical application platform based on 3D Slicer. For details, please refer to the Supplementary Materials.

## Data Availability

Deidentified data will be available on reasonable request and approval by the first author, the corresponding author, and all coauthors of the study.
