# Supplementary material for: A New Fluorine-18-Fluoroethyltyrosine Positron-Emission-Tomography/Magnetic-Resonance-Based Tumor Resection Plan Improved the Prognosis of Glioblastoma Patients. A Multicenter Validated Study
Source: Research (Wash D C). 2026 Aug 4;9:1335. doi: 10.34133/research.1335 (PMC13434045; doi:10.34133/research.1335)
Supplement: Supplementary file 1 [file research.1335.f1.zip › Revised Supplementary Methods with track changes.docx]

**Hybrid ^18^F-FET PET/CE MRI imaging**

The ^18^F-FET PET acquisition protocol was broadly performed in accordance with the current joint EANM/EANO/RANO/SNMMI practice guideline for glioma imaging [17]. Patients were scanned on one of two PET/MR systems—GE Signa (GE Healthcare, USA; n=177) or uPMR 790 (United Imaging, China; n=62). (1) GE Signa: All patients were injected intravenously [18F]FET at the recommended activities for brain imaging in adults (185–200 MBq) while remaining fasted for more than 4 h. The PET static images of brain were acquired from 20 to 40 min with multi-parametric MR scanning simultaneously following the [^18^F]FET injection. And 3.0-T MR scanning of brain initially performed a Dixon MR sequence used to process the attenuation correction of PET images followed by other multi-parametric 3.0-T MR scanning included an axial T1-weighted fluid-attenuated inversion recovery (T1 FLAIR) sequence, an axial T2-weighted (T2) sequence, an T2-weighted fluid-attenuated inversion recovery (T2 FLAIR) sequence, an axial diffusion weighted imaging (DWI) sequence, sequence, a (3D) T1 sequence, and a 3D contrast-enhanced T1-weighted sequence (3D T1CE). The specific MRI parameters included FLAIR (repetition time (TR)/ echo time (TE)=6200/140 ms, slice=22, voxel size=1.00×1.00×5.00 mm^3^ ), DWI (TR/TE=5000/Minimum ms, with b values of 1000s/ mm^2^ , slice=44, voxel size=1.30×1.30×5.00 mm^3^ ) and 3D T1-CE (TR/TE=2100/Minimum ms, slice=188, voxel size=1.00×1.00×1.00 mm^3^ ). The apparent diffusion coefficient (ADC) map was generated from the DWI data via the AW 4.7 workstation (GE Health care). The 3D TOF ordered-subset expectation maximization (OSEM) algorithm (6 iterations and 16 subsets) was used to reconstruct PET images with the field of view, matrix, and slice thickness set to 35 cm, 256 × 256 (voxel size 1.36 × 1.36 mm) and 2.78 mm, respectively. And brain motion correction technique based on the line of response (LOR) was used in PET image reconstruction. (2) uPMR 790: [^18^F]FET at the recommended activities for brain imaging in adults (185–200 MBq) while remaining fasted for more than 4 h. Subsequently, a brain PET/MR scan was initiated simultaneously. Initially, the images obtained from water-fat imaging (WFI) were processed to eliminate any bias field signals. Following this correction, the images underwent a segmentation process where they were classified into four distinct categories based on their specific attenuation coefficients. These categories included soft tissue, fat, lung and air. Raw data was reconstructed on the console. The PET data were reconstructed with a combination of ordered subsets expectation maximisation, time-of-flight and point spread function methods. The reconstruction parameters included two iterations and 20 subsets for the four different methods. The PET images were reconstructed with a full width at half maximum of a Gaussian filter of 3.0mm and were reconstructed into a 192×192 matrix. The voxel size was 1.82×1.82 × 2.78mm^3^. During PET acquisition, diagnostic sequences commonly used in clinical practices such as coronal three-dimensional T1-weighted, T2-weighted, T2 FLAIR images and diffusion weighted imaging (DWI) sequence were also scanned to identify specific lesion locations. The total imaging time was 20min. A coronal plane acquisition of a 3D T1-weighted spoiled gradient-echo sequence was conducted, using Dixon-based WFI for separation,26 with the following specifications: repetition time (TR)=4.91 ms, echo time (TE)=2.24 ms, flip angle (FA)=10°, field of view (FOV)=500 × 350mm, matrix size=549 × 384, slice thickness=2mm. T2-weighted and T2 FLAIR images were acquired for lesion identification with the following parameters: T2-weighted images: TR=5000, TE=Minimum, FOV=240 × 240mm, matrix size=288 × 288, slice thickness=4mm. T2 FLAIR: TR=7000 ms, TE=79 ms, inversion time=2500 ms, FA=120°, FOV=240 × 240mm, matrix size=256 × 192, slice thickness=4mm. DWI with the following parameters: TR=5000, TE=Minimum, FOV=240 × 240mm, matrix size=128 × 128, slice thickness=4mm.

**^18^F-FET PET cross-modal tumor delineation system**

All acquired images were reviewed by experienced radiologists after scanning to ensure image quality. CE MRI and ^18^F-FET PET images were treated as reference and moving images, respectively. Due to their inherent differences in modality, we employed Symmetric Normalization with Robust Average (SyNRA) for deformable registration. SyN is a diffeomorphic non-rigid registration method that optimizes deformation fields to align images, particularly effective for large deformations. SyNRA utilizes a region-based robust average intensity difference as the similarity metric, demonstrating strong robustness against inter-modal intensity variations. The registration process was implemented using the Advanced Normalization Tools (ANTs) package, ensuring precise spatial correspondence between the metabolic boundaries derived from ^18^F-FET PET and the anatomical structures identified in CE MRI.

Tumor delineation was performed on the registered PET images using a clinically validated threshold-based approach, rather than an independently trained AI segmentation model. Specifically, the PET-defined tumor volume (VPET) was generated using a tumor-to-background ratio (TBR) threshold of 1.6, in accordance with current guideline recommendations and our previously published biopsy-validated hybrid PET/MR workflow described by Song et al. [15]. Background activity was measured in the contralateral normal-appearing hemisphere using reference regions as previously described.

The initial binary tumor mask was refined using morphological operations, including opening and closing, followed by region filtering to reduce noise and exclude obvious non-tumorous regions of interest. After this semi-automatic threshold-based segmentation, the contours were reviewed and manually corrected by two experienced nuclear medicine radiologists/physicians, who removed vascular structures and other non-tumorous regions when necessary. Final hole-filling was then performed to generate a continuous tumor mask, and the resulting contours were projected onto MRI for visualization.

The workflow was implemented using the Open Source Computer Vision Library (OpenCV) for image processing and integrated into 3D Slicer for interactive visualization and surgical navigation. Thus, in the present study, the system functioned primarily as a computer-assisted cross-modal delineation and navigation-integration platform, rather than as a fully automated AI model for independent tumor boundary determination.

**Volume and TBR analysis**

The V_CE_ was obtained from preoperative CE T1-weighted MRI, and the V_FLAIR_ was obtained from preoperative FLAIR images. In the ^18^F-FET PET group, the V_PET_ was delineated on co-registered static ^18^F-FET PET images using a TBR threshold of 1.6, following the methodological framework and current joint practice recommendations for amino-acid PET in glioma imaging. Spatial overlap between V_CE_ and the whole V_FET_ was quantified using the Dice score. TBRmax and TBRmean were extracted for both the CE MRI-defined contrast-enhancing region and the ^18^F-FET PET-defined whole metabolically active region in the ^18^F-FET PET group. Tumor volumes were measured in cm³.

**Intraoperatively Verification of Tumor Boundary**

To validate the biological heterogeneity between tumor boundaries delineated by CE MRI and ^18^F-FET PET, three complementary approaches were employed including: (1) Ultramicroscopic Cellular Imaging: Ultramicroscopic cellular imaging was used intraoperatively to assess the presence of residual tumor cells at the resection cavity margin.. (2) iMRI: During surgery, iMRI was used in real time to repeatedly confirm tumor margins and ensure that resection conformed to the preoperatively defined boundaries. This helped to visualize residual CE tissue and refine the surgical plan dynamically. (3) Intraoperative ultrasound (IoUS): Concurrently, intraoperative ultrasound was applied to localize the tumor and verify the anatomical boundaries delineated by both imaging modalities. Real-time ultrasound feedback was particularly useful in identifying hyperechoic tumor tissue and assessing depth of invasion in eloquent areas. Owing to hardware constraints, the intraoperatively verification were conducted exclusively at two participating centers (Xuanwu and Huashan).

**Histopathological pathology**

Tissue samples were collected intraoperatively from tumor boundary regions as defined by CE MRI and ^18^F-FET PET, respectively. Each sample was immediately fixed in 10% neutral-buffered formalin for 24-48 hours, followed by standard paraffin embedding. Sections of 4-μm thickness were prepared using a microtome and stained with hematoxylin and eosin (HE). Histological evaluation was independently performed by at least two board-certified neuropathologists at each participating center who were blinded to the surgical group assignment and had no conflicts of interest with this study. Parameters assessed included: tumor cellularity, nuclear pleomorphism, mitotic activity, microvascular proliferation, and the presence or absence of necrosis or reactive gliosis. Tumor infiltration was graded semi-quantitatively as "none," "focal," or "diffuse." Lymphocytic infiltrates and microglial activation were noted when present. Discrepancies between reviewers were resolved by consensus.

**Immunohistochemistry**

Immunohistochemical staining was performed on adjacent sections obtained from formalin-fixed, paraffin-embedded (FFPE) tumor tissues resected from the proliferative region and the boundary infiltrative region. The following primary antibodies were used: Ki67 (1:200, Dako M7240) for assessing proliferative activity, CD31 (1:100, Abcam ab28364) for evaluating microvessel density, and SOX2 (1:200, Abcam ab97959) as a marker of stemness. Antigen retrieval was conducted in citrate buffer (pH 6.0) using microwave heating, and endogenous peroxidase activity was blocked with 3% hydrogen peroxide. Tissue sections were incubated with primary antibodies overnight at 4 ℃, followed by visualization using a biotin-streptavidin-peroxidase system with DAB as chromogen. Hematoxylin was used for nuclear counterstaining. Stained sections were imaged under a light microscope at 400x magnification. For each marker, five randomly selected high-power fields per region were analyzed per case. Quantitative analysis was conducted using Image-Pro Plus software to calculate the IOD of positively stained areas. Mean IOD values were used for statistical comparison between the two regions.

**Extent of resection assessment according to the RANO resect classifier**

In addition to the intraoperative outcome assessment, the extent of resection was evaluated according to the RANO resect classification [21]. Based on postoperative residual tumor burden, patients were categorized as Class 1, Class 2A, Class 2B, Class 3A, Class 3B, or Class 4. The distribution of RANO resect categories between the CE MRI and 18F-FET PET groups was compared using Fisher’s exact test. In addition, a simplified sensitivity analysis was conducted by collapsing the RANO resect classifier into two categories (Class 1 vs. ≥Class 2A), and the resulting proportions were compared between the CE MRI and 18F-FET PET groups using the Chi-squared test.

**Single-cell RNA Sequencing**

Fresh tumor boundary tissues were collected intraoperatively from regions defined by CE MRI and ^18^F-FET PET, respectively. Three representative patients were selected for each group (n = 6 total), and tissue samples were immediately transferred into ice-cold MACS Tissue Storage Solution (Miltenyi Biotec) and processed within 30 minutes post-excision. Samples were minced and enzymatically dissociated using the Tumor Dissociation Kit (Miltenyi Biotec) following the manufacturer’s protocol. Mechanical dissociation was aided by the gentle MACS Dissociator. After filtration through a 70 μm cell strainer, cells were centrifuged and resuspended in PBS with 0.04% BSA. Cell viability was confirmed using trypan blue exclusion (>85% required). single-cell RNA sequencing (scRNA-seq) suspensions were loaded onto a 10x Genomics Chromium Controller using the Chromium Next GEM Single Cell 3' v3.1 kit. Target capture was ~6,000 cells per sample. Libraries were constructed following the 10x Genomics protocol and sequenced on an Illumina NovaSeq 6000 platform (paired-end 150 bp) to achieve a minimum depth of 50,000 reads per cell. Raw sequencing data were processed using the Cell Ranger pipeline (v6.1.2) aligned to the human GRCh38 genome. Downstream analysis was conducted using Seurat (v4.1.3). Low-quality cells (genes <200 or >5,000; mitochondrial gene content >15%) were excluded. Data were normalized using SCTransform, followed by dimensionality reduction (PCA, UMAP), clustering (Louvain algorithm), and differential expression analysis. Cell types were annotated based on canonical markers. Proportional composition and differentially expressed gene sets were compared between CE MRI and ^18^F-FET PET defined boundary regions.

**Assessments and Outcomes**

All patients included in this study were followed prospectively for clinical outcomes and quality of life, including OS, PFS and QoL. Cranial CE MRI scans were performed at three, six, nine, and 12 months postoperatively, along with outpatient clinical review. QoL was assessed using the EORTC QLQ-C30 (core cancer module) and EORTC QLQ-BN20 (brain tumor module). Assessments were performed at three, six, nine, and 12 months postoperatively. At each timepoint, patients were also scheduled for routine clinical evaluation and follow-up cranial CE MRI to assess for radiological progression or recurrence. Questionnaires were administered in person or electronically and reviewed for completeness by study coordinators.

**Statistical Analysis**

All statistical analyses were performed using R (version 4.1.3) with the packages WeightIt, survey, and so on. Baseline characteristics between the CE MRI and ^18^F-FET PET groups were first compared before weighting using Student’s t test for normally distributed continuous variables, the Wilcoxon rank-sum test for non-normally distributed variables, and the chi-square or Fisher’s exact test for categorical variables. standardized mean differences (SMDs) were also calculated to quantify group imbalances.

To adjust for confounding, IPTW was implemented using propensity scores estimated from a logistic regression model including age, sex, tumor size, tumor laterality, *MGMT* promoter methylation status, *TERT* promoter mutation status, *EGFR* amplification status, Ki67 index, and pre- and postoperative KPS scores. Stabilized weights were applied to construct weighted cohorts. After weighting, baseline balance between groups was primarily assessed using SMDs, while weighted sample sizes (weighted n, i.e., the sum of weights in each group) were reported for descriptive purposes.

For QoL outcomes (EORTC QLQ-C30 and QLQ-BN20), weighted mean scores and 95% CIs were estimated at three, six, nine, and 12 months postoperatively using survey-weighted linear regression models (svyglm in the survey package). Between-group differences were reported as regression coefficients (mean difference between CE MRI and ^18^F-FET PET groups) with corresponding 95% CIs and *p* values. The *p* < 0.05 was considered statistically significant. OS and PFS will be estimated using the KM method, and survival curves will be plotted accordingly.

**Role of the funding source**

The funders had no role in study design, data collection, data analysis, data interpretation, or writing or decision for publication of the report.
