# Supplementary material for: A New Fluorine-18-Fluoroethyltyrosine Positron-Emission-Tomography/Magnetic-Resonance-Based Tumor Resection Plan Improved the Prognosis of Glioblastoma Patients. A Multicenter Validated Study
Source: Research (Wash D C). 2026 Aug 4;9:1335. doi: 10.34133/research.1335 (PMC13434045; doi:10.34133/research.1335)
Supplement: Supplementary file 1 [file research.1335.f1.zip › Revised Tables S1 to S8 with track changes.docx]

**Tables S1. Characteristics of the participants**

| **Center** | **XW** | **E** | **HS** | **MZP** | **TJ** |
| --- | --- | --- | --- | --- | --- |
| **Total(N=239)** | 204 | 7 | 6 | 17 | 5 |
| **Age, year** | 58·00(48·00, 65·00) | 69·00(59·00, 74·00) | 55·50(43·75, 64·00) | 58·00(47·00, 66·50) | 60·00(55·00, 75·00) |
| **Sex** |  |  |  |  |  |
| Female | 85 | 4 | 5 | 9 | 3 |
| Male | 119 | 3 | 1 | 8 | 2 |

XW = Xuanwu Hospital Capital Medical University; E = East Hospital Affiliated to Tongji University; HS = Huashan Hospital Fudan University; MZP = Meizhou People’s Hospital, Meizhou Academy of Medical Sciences; TJ = Tongji Hospital, Tongji Medical College of HUST

^18^F-FET = ^18^F-fluoroethyltyrosine

**Tables S2. Comparison of baseline demographic, clinical, and molecular characteristics between the CE MRI and ^18^F-FET PET groups**

| **Characteristics (mean (SD))** | **CE MRI (n = 124)** | **^18^F-FET PET (n = 115)** | ***p*** |
| --- | --- | --- | --- |
| **V_CE_ (cm^3^)** | 39·21 [25·01, 49·18] | 39·63 [26·33, 48·78] | 0·84^7^ |
| **V_FLAIR_ (cm^3^)** | 96·03 [72·15, 116·53] | 94·96 [78·81, 127·50] | 0·24^7^ |
| **V_CE_** *vs* **V_PET_ (cm^3^)** | 39·21 [25·01, 49·18] | 84·65 [65·31, 105·92] | < 0·001^7^ |
| **Dice score** | / | 0.58 ± 0.11 | / |
| **Age (years)** | 52·00 [44·75, 57·00] | 50·00 [43·00, 55·50] | 0·26^7^ |
| **Preoperative KPS** | 95·00 [90·00, 100·00] | 95·00 [90·00, 100·00] | 0·83^7^ |
| **Postoperative KPS** | 65·00 [35·00, 90·00] | 70·00 [35·00, 90·00] | 0·76^7^ |
| **Ki67** | 40·00 [30·00, 60·00] | 40·00 [30·00, 50·00] | 0·93^7^ |
| **Side** | L: 67 (54·0%); R: 57 (46·0%) | L: 67 (58·3%); R: 48 (41·7%) | 0·60^8^ |
| **Gender** | f: 59 (47·6%); m: 65 (52·4%) | f: 46 (40·0%); m: 69 (60·0%) | 0·29^8^ |
| **MGMT** | 0^1^: 74 (59·7%); 1^2^: 50 (40·3%) | 0^1^: 76 (66·1%); 1^2^: 39 (33·9%) | 0·37^8^ |
| **TERT** | 0^3^: 36 (29·0%); 1^4^: 88 (71·0%) | 0^3^: 30 (26·1%); 1^4^: 85 (73·9%) | 0·72^8^ |
| **EGFR** | 0^5^: 48 (38·7%); 1^6^: 76 (61·3%) | 0^5^: 42 (36·5%); 1^6^: 73 (63·5%) | 0·83^8^ |

^1^Unmethylated MGMT promoter, ^2^MGMT promoter methylation

^3^TERT promoter wild-type, ^4^TERT promoter mutations

^5^EGFR wild-type, ^6^EGFR amplification

^7^Wilcoxon; ^8^Chi-squared

CE MRI = Contrast Enhanced Magnetic Resonance Imaging; ^18^F-FET = ^18^F-fluoroethyltyrosine; IPTW = Inverse Probability of Treatment Weighting; SMD = Standardized mean difference; L = Left; R = Right; F = Female; M = Male; TBR = tumor-to-background ratio

**Tables S3. Comparison of TBR in ^18^F-FET PET group**

|  | **CE MRI (n = 115)** | **^18^F-FET PET (n = 115)** | ***p*** |
| --- | --- | --- | --- |
| **TBRmax** | 4·14 [3·63, 4·74] | 4·14 [3·63, 4·74] | 1·00 |
| **TBRmean** | 2·68 [2·25, 3·11] | 2·60 [2·23, 3·03] | 0·65 |

Wilcoxon

CE MRI = Contrast Enhanced Magnetic Resonance Imaging; ^18^F-FET = ^18^F-fluoroethyltyrosine; TBR = tumor-to-background ratio

**Tables S4. Comparison of baseline demographic, clinical, and molecular characteristics between the CE MRI and ^18^F-FET PET groups after IPTW**

| **Characteristics (mean (SD)/%)** | **CE MRI (Weighted n = 123·78)** | **^18^F-FET PET (Weighted n = 114·70)** | **test SMD** |
| --- | --- | --- | --- |
| **V_CE_ (cm^3^)** | 42·78 (22·04) | 42·99 (26·87) | 0·008 |
| **V_FLAIR_ (cm^3^)** | 101·73 (40·70) | 102·93 (44·73) | 0·028 |
| **Age (years)** | 49·29 (8·47) | 49·16 (8·38) | 0·016 |
| **Ki67** | 42·06 (19·04) | 42·28 (18·71) | 0·012 |
| **Preoperative KPS** | 92·69 (9·43) | 92·73 (9·51) | 0·004 |
| **Postoperative KPS** | 60·08 (29·32) | 60·41 (29·54) | 0·011 |
| **Side** | L: 69·9 (56·4); R: 53·9 (43·6) | L: 64·9 (56·6); R: 49·8 (43·4) | 0·003 |
| **Gender** | F: 54·0 (43·7); M: 69·7 (56·3) | F: 50·3 (43·9); M: 64·4 (56·1) | 0·025 |
| **MGMT** | 0^1^: 77·2 (64·0); 1^2^: 46·6 (37·7) | 0^1^: 71·6 (62·4); 1^2^: 43·1 (37·6) | 0·002 |
| **TERT** | 0^3^: 33·4 (27·0); 1^4^: 90·4 (73·0) | 0^3^: 31·0 (27·0); 1^4^: 87·3 (73·0) | 0·001 |
| **EGFR** | 0^5^: 46·3 (37·4); 1^6^: 77·5 (62·6) | 0^5^: 42·7 (37·2); 1^6^: 72·0 (62·8) | 0·004 |

^1^Unmethylated MGMT promoter, ^2^MGMT promoter methylation

^3^TERT promoter wild-type, ^4^TERT promoter mutations

^5^EGFR wild-type, ^6^EGFR amplification

CE MRI = Contrast Enhanced Magnetic Resonance Imaging; 18F-FET = ^18^F-fluoroethyltyrosine; IPTW = Inverse Probability of Treatment Weighting; SD = Standard deviation; SMD = Standardized mean difference; L = Left; R = Right; F = Female; M = Male; TBR = tumor-to-background ratio

**Tables S5. Unadjusted comparison of EORTC QLQ-C30 scores between CE MRI and ^18^F-FET PET groups at three, six, nine, and 12 months post-surgery**

|  | **3 months** | | ***p*** | **6 months** | | ***p*** | **9 months** | | ***p*** | **12 months** | | ***p*** |
| --- | --- | --- | --- | --- | --- | --- | --- | --- | --- | --- | --- | --- |
|  | **CE MRI (n = 120)** | **^18^F-FET PET (n = 114)** |  | **CE MRI (n = 109)** | **^18^F-FET PET (n = 106)** |  | **CE MRI (n = 91)** | **^18^F-FET PET (n = 99)** |  | **CE MRI (n = 83)** | **^18^F-FET PET (n = 91)** |  |
| **Physical Functioning(Q1-5)** | 78·71 ± 4·83 | 79·54 ± 4·65 | 0·18^1^ | 78·75 ± 4·88 | 79·79 ± 4·57 | 0·11^1^ | 78·75 ± 4·73 | 79·48 ± 4·44 | 0·27^1^ | 78·72 ± 4·82 | 79·32 ± 4·45 | 0·40^1^ |
| **Role Functioning(Q6-7)** | 80·11 ± 4·59 | 79·52 ± 4·26 | 0·30^1^ | 80·21 ± 4·72 | 79·55 ± 4·25 | 0·28^1^ | 80·36 ± 4·78 | 79·43 ± 4·35 | 0·16^1^ | 80·36 ± 4·70 | 79·35 ± 4·38 | 0·16^1^ |
| **Cognitive Functioning(Q8-9)** | 76·00 (71·00, 81·00) | 75·50 (71·25, 81·75) | 0·49^2^ | 76·00 (72·00, 81·00) | 77·00 (71·00, 82·00) | 0·79^2^ | 76·00 (72·00, 81·00) | 76·00 (71·00, 81·50) | 0·79^2^ | 75·41 ± 6·51 | 76·04 ± 6·54 | 0·52^1^ |
| **Emotional Functioning(10-13)** | 74·75 ± 5·94 | 75·79 ± 5·37 | 0·16^1^ | 75·16 ± 5·70 | 75·70 ± 5·43 | 0·48^1^ | 75·11 ± 5·76 | 75·96 ± 5·30 | 0·29^1^ | 75·65 ± 5·48 | 76·04 ± 5·21 | 0·63^1^ |
| **Social Functioning(Q14-15)** | 75·08 ± 6·63 | 76·70 ± 5·88 | 0·05^1^ | 75·02 ± 6·79 | 76·71 ± 5·98 | 0·05^1^ | 75·07 ± 6·86 | 76·56 ± 5·93 | 0·11^1^ | 75·00 (70·00, 79·50) | 76·00 (74·00, 80·50) | 0·16^2^ |
| **Fatigue(Q16-18)** | 26·22 ± 7·91 | 24·69 ± 7·30 | 0·13^1^ | 26·50 ± 7·79 | 24·83 ± 7·41 | 0·11^1^ | 26·76 ± 7·93 | 25·16 ± 7·48 | 0·16^1^ | 27·08 ± 7·69 | 25·33 ± 7·49 | 0·13^1^ |
| **Pain(Q19-20)** | 9·00 (8·00, 10·00) | 9·00 (8·00, 9·00) | 0·73^2^ | 9·00 (8·00, 10·00) | 9·00 (8·00, 9·75) | 0·99^2^ | 9·00 (8·00, 10·00) | 9·00 (8·00, 10·00) | 0·85^2^ | 9·00 (8·00, 10·00) | 9·00 (8·00, 10·00) | 0·9335^2^ |
| **Nausea and Vomiting(Q20-21)** | 8·00 (5·00, 10·00) | 7·00 (4·00, 10·00) | 0·19^2^ | 7·00 (5·00, 10·00) | 7·00 (4·00, 10·00) | 0·31^2^ | 8·00 (5·00, 10·00) | 7·00 (4·00, 10·00) | 0·32^2^ | 7·00 (5·00, 10·00) | 7·00 (4·00, 10·00) | 0·39^2^ |
| **Dyspnea(Q23)** | 5·00 (3·00, 10·00) | 4·00 (3·00, 7·00) | 0·22^2^ | 5·00 (3·00, 10·00) | 4·00 (3·00, 6·00) | 0·15^2^ | 5·00 (3·00, 10·00) | 4·00 (3·00, 6·00) | 0·1^2^ | 5·00 (3·00, 10·00) | 4·00 (3·00, 6·00) | 0·10^2^ |
| **Sleep disturbance(Q24)** | 13·00 (10·00, 19·00) | 15·00 (10·00, 18·75) | 0·18^2^ | 13·00 (10·00, 18·00) | 15·00 (10·00, 18·75) | 0·14^2^ | 13·00 (10·00, 18·50) | 15·00 (10·00, 18·50) | 0·23^2^ | 13·00 (10·00, 19·00) | 15·00 (10·00, 18·00) | 0·32^2^ |
| **Appetite loss(Q25)** | 19·00 (15·00, 22·00) | 20·00 (15·00, 24·00) | 0·20^2^ | 19·00 (16·00, 22·00) | 20·00 (15·00, 24·00) | 0·27^2^ | 19·03 ± 5·14 | 19·21 ± 5·27 | 0·81^1^ | 19·12 ± 5·05 | 18·98 ± 5·30 | 0·86^1^ |
| **Constipation(Q26)** | 9·00 (5·00, 12·00) | 9·00 (6·00, 13·00) | 0·24^2^ | 8·00 (5·00, 12·00) | 9·50 (7·00, 14·00) | 0·04^2^ | 8·00 (5·00, 12·00) | 10·00 (6·00, 14·00) | 0·07^2^ | 9·00 (5·00, 12·50) | 10·00 (7·00, 14·00) | 0·14^2^ |
| **Diarrhea(Q27)** | 4·00 (1·75, 5·25) | 4·00 (2·00, 6·00) | 0·37^2^ | 4·00 (2·00, 6·00) | 4·00 (2·00, 6·00) | 0·58^2^ | 4·00 (2·00, 6·00) | 4·00 (2·00, 6·00) | 0·48^2^ | 4·00 (1·50, 6·00) | 4·00 (2·00, 6·00) | 0·45^2^ |
| **Financial difficulties(Q28)** | 38·51 ± 6·26 | 39·54 ± 6·33 | 0·21^1^ | 38·47 ± 6·26 | 39·84 ± 6·31 | 0·11^1^ | 38·62 ± 6·22 | 39·88 ± 6·40 | 0·17^1^ | 38·61 ± 6·20 | 40·02 ± 6·22 | 0·13^1^ |
| **Global Health Status(Q29-30)** | 77·00 (76·00, 78·25) | 77·00 (75·00, 78·00) | 0·13^2^ | 77·00 (76·00, 78·00) | 76·50 (75·00, 78·00) | 0·20^2^ | 77·00 (76·00, 79·00) | 76·00 (75·00, 78·00) | 0·13^2^ | 77·23 ± 2·76 | 76·69 ± 2·65 | 0·19^1^ |

^1^t-test; ^2^Wilcoxon

CE MRI = Contrast Enhanced Magnetic Resonance Imaging; ^18^F-FET = ^18^F-fluoroethyltyrosine ; IPTW = Inverse Probability of Treatment Weighting; EORTC = European Organisation for Research and Treatment of Cancer; QLQ-C30 = Quality of Life Questionnaire-Core 30; QLQ-BN20 = Quality of Life Questionnaire-Brain Neoplasm Module

**Tables S6. Unadjusted comparison of EORTC QLQ-BN20 scores between CE MRI and ^18^F-FET PET groups at three, six, nine, and 12 months post-surgery**

|  | **3 months** | | ***p*** | **6 months** | | ***p*** | **9 months** | | ***p*** | **12 months** | | ***P*** |
| --- | --- | --- | --- | --- | --- | --- | --- | --- | --- | --- | --- | --- |
|  | **CE MRI (n = 120)** | **^18^F-FET PET (n = 114)** |  | **CE MRI (n = 109)** | **^18^F-FET PET (n = 106)** |  | **CE MRI (n = 91)** | **^18^F-FET PET (n = 99)** |  | **CE MRI (n = 83)** | **^18^F-FET PET (n = 91)** |  |
| **Future Uncertainty(Q1-4)** | 79·00 (75·75, 83·00) | 81·00 (76·00, 85·00) | 0·09^2^ | 79·00 (75·00, 83·00) | 80·00 (76·00, 85·00) | 0·30^2^ | 79·00 (75·00, 83·00) | 80·00 (76·00, 85·00) | 0·27^2^ | 79·00 (75·50, 83·00) | 80·00 (76·00, 84·00) | 0·58^2^ |
| **Visual Disorder(Q6-8)** | 9·00 (7·00, 11·25) | 9·00 (6·00, 12·00) | 0·33^2^ | 9·00 (7·00, 12·00) | 9·00 (6·00, 12·00) | 0·33^2^ | 9·00 (7·00, 12·00) | 8·00 (6·00, 12·00) | 0·16^2^ | 9·00 (7·00, 12·00) | 8·00 (6·00, 11·50) | 0·07^2^ |
| **Motor Dysfunction(Q10、15、19)** | 54·13 ± 12·33 | 51·07 ± 14·78 | 0·09^1^ | 54·03 ± 12·64 | 51·65 ± 14·78 | 0·21^1^ | 55·19 ± 12·65 | 51·84 ± 15·12 | 0·10^1^ | 54·82 ± 12·90 | 51·46 ± 14·94 | 0·11^1^ |
| **Communication Deficit(Q11-13)** | 17·00 (8·75, 25·00) | 19·00 (11·00, 28·00) | 0·13^2^ | 16·00 (8·00, 25·00) | 18·50 (10·00, 27·75) | 0·26^2^ | 18·00 (9·50, 25·00) | 18·00 (10·00, 28·00) | 0·49^2^ | 15·00 (8·00, 25·00) | 18·00 (9·50, 28·00) | 0·31^2^ |
| **Social Functioning(Q14-15)** | 71·39 ± 9·45 | 69·32 ± 10·51 | 0·12^1^ | 71·63 ± 9·40 | 69·79 ± 10·33 | 0·17^1^ | 71·66 ± 9·47 | 69·05 ± 10·03 | 0·07^1^ | 71·65 ± 9·14 | 68·86 ± 10·08 | 0·06^1^ |
| **Seizures(Q9)** | 5·00 (2·75, 10·00) | 4·00 (3·00, 7·00) | 0·45^2^ | 6·00 (3·00, 10·00) | 4·00 (3·00, 7·00) | 0·39^2^ | 6·00 (3·00, 10·00) | 4·00 (3·00, 7·00) | 0·41^2^ | 5·00 (2·50, 10·00) | 5·00 (3·00, 7·00) | 0·73^2^ |
| **Drowsiness(Q14)** | 16·50 (12·00, 20·00) | 18·00 (13·00, 21·75) | 0·12^2^ | 17·00 (12·00, 20·00) | 18·00 (13·00, 21·00) | 0·14^2^ | 16·00 (12·00, 19·50) | 18·00 (12·50, 21·00) | 0·13^2^ | 16·00 (12·00, 19·50) | 17·00 (12·00, 21·00) | 0·24^2^ |
| **Hair Loss(Q16)** | 11·00 (9·00, 14·00) | 11·00 (8·25, 14·00) | 0·64^2^ | 11·00 (9·00, 14·00) | 12·00 (8·25, 15·00) | 0·58^2^ | 11·30 ± 4·05 | 11·68 ± 4·08 | 0·52^1^ | 11·16 ± 4·03 | 11·53 ± 4·16 | 0·55^1^ |
| **Itchy Skin(Q17)** | 6·00 (1·00, 11·00) | 3·00 (1·00, 8·00) | 0·25^2^ | 6·00 (1·00, 11·00) | 3·50 (1·00, 8·00) | 0·27^2^ | 6·00 (1·00, 11·00) | 3·00 (1·00, 8·00) | 0·11^2^ | 6·00 (1·00, 11·00) | 3·00 (1·00, 8·00) | 0·26^2^ |
| **Weakness of Legs(Q18)** | 14·00 (13·00, 20·00) | 13·00 (13·00, 26·00) | 0·46^2^ | 14·00 (13·00, 21·00) | 13·50 (13·00, 26·00) | 0·47^2^ | 14·00 (13·00, 22·50) | 14·00 (13·00, 26·00) | 0·90^2^ | 14·00 (13·00, 22·50) | 15·00 (13·00, 28·50) | 0·62^2^ |
| **Bladder Control(Q20)** | 3·00 (0·00, 10·00) | 3·00 (1·00, 8·00) | 0·68^2^ | 3·00 (0·00, 10·00) | 3·00 (1·00, 8·00) | 0·62^2^ | 3·00 (0·00, 11·00) | 3·00 (1·00, 8·00) | 0·71^2^ | 2·00 (0·00, 10·00) | 3·00 (1·00, 8·00) | 0·71^2^ |
| **Feel unsteady on feet(Q19)** | 17·00 (9·00, 26·25) | 16·00 (9·00, 22·75) | 0·20^2^ | 17·00 (9·00, 28·00) | 16·00 (9·00, 23·00) | 0·23^2^ | 18·00 (9·00, 26·50) | 16·00 (8·50, 23·00) | 0·28^2^ | 18·00 (9·00, 26·00) | 16·00 (8·00, 22·50) | 0·21^2^ |

CE MRI = Contrast Enhanced Magnetic Resonance Imaging; ^18^F-FET = ^18^F-fluoroethyltyrosine ; IPTW = Inverse Probability of Treatment Weighting; EORTC = European Organisation for Research and Treatment of Cancer; QLQ-C30 = Quality of Life Questionnaire-Core 30; QLQ-BN20 = Quality of Life Questionnaire-Brain Neoplasm Module

^1^t-test; ^2^Wilcoxon

**Tables S7. IPTW-adjusted comparison of EORTC QLQ-C30 scores between CE MRI and ^18^F-FET PET groups at three, six, nine, and 12 months post-surgery**

|  | **3 months** | | ***p*** | **6 months** | | ***p*** | **9 months** | | ***p*** | **12 months** | | ***p*** |
| --- | --- | --- | --- | --- | --- | --- | --- | --- | --- | --- | --- | --- |
|  | **CE MRI ( Weighted n = 120·65)** | **^18^F-FET PET (Weighted n = 113·34)** |  | **CE MRI (Weighted n = 109·42)** | **^18^F-FET PET (Weighted n = 105·31)** |  | **CE MRI (Weighted n = 91·34)** | **^18^F-FET PET (Weighted n = 98·91)** |  | **CE MRI (Weighted n = 83·03)** | **^18^F-FET PET (Weighted n = 91·15)** |  |
| **Physical Functioning(Q1-5)** | 78·8 (77·9–79·6) | 79·6 (78·7–80·4) | 0·24 | 78·8 (77·9–79·7) | 79·8 (78·9–80·7) | 0·14 | 78·8 (77·9–79·8) | 79·5 (78·6–80·3) | 0·34 | 78·8 (77·8–79·8) | 79·3 (78·4–80·2) | 0·47 |
| **Role Functioning(Q6-7)** | 80·0 (79·2–80·8) | 79·5 (78·7–80·3) | 0·39 | 80·1 (79·2–80·9) | 79·5 (78·7–80·3) | 0·37 | 80·2 (79·2–81·2) | 79·4 (78·5–80·3) | 0·22 | 80·2 (79·2–81·2) | 79·3 (78·4–80·2) | 0·20 |
| **Cognitive Functioning(Q8-9)** | 75·3 (74·1–76·6) | 76·0 (74·8–77·2) | 0·44 | 75·8 (74·5–77·0) | 76·0 (74·8–77·3) | 0·77 | 75·6 (74·2–77·0) | 75·9 (74·6–77·2) | 0·77 | 75·4 (74·0–76·8) | 76·1 (74·7–77·4) | 0·51 |
| **Emotional Functioning(10-13)** | 74·6 (73·6–75·7) | 75·9 (74·9–76·9) | 0·08 | 75·0 (73·9–76·0) | 75·8 (74·8–76·9) | 0·26 | 74·9 (73·7–76·1) | 76·1 (75·0–77·1) | 0·15 | 75·4 (74·2–76·6) | 76·2 (75·1–77·2) | 0·35 |
| **Social Functioning(Q14-15)** | 75·2 (74·0–76·4) | 76·6 (75·5–77·7) | 0·08 | 75·1 (73·9–76·4) | 76·6 (75·5–77·8) | 0·09 | 75·1 (73·7–76·5) | 76·5 (75·3–77·7) | 0·15 | 75·0 (73·4–76·6) | 76·5 (75·3–77·7) | 0·13 |
| **Fatigue(Q16-18)** | 26·1 (24·7–27·6) | 24·7 (23·4–26·0) | 0·15 | 26·5 (25·0–27·9) | 24·8 (23·4–26·2) | 0·12 | 26·7 (25·1–28·4) | 25·1 (23·7–26·6) | 0·15 | 27·1 (25·4–28·8) | 25·3 (23·7–26·8) | 0·12 |
| **Pain(Q19-20)** | 8·7 (8·5–9·0) | 8·6 (8·4–8·9) | 0·69 | 8·7 (8·4–9·0) | 8·7 (8·4–8·9) | 0·97 | 8·7 (8·4–9·0) | 8·7 (8·4–9·0) | 0·90 | 8·7 (8·4–9·0) | 8·7 (8·5–9·0) | 0·84 |
| **Nausea and Vomiting(Q20-21)** | 7·5 (6·9–8·1) | 6·8 (6·2–7·4) | 0·10 | 7·5 (6·8–8·1) | 6·8 (6·2–7·5) | 0·18 | 7·5 (6·8–8·2) | 6·8 (6·1–7·5) | 0·18 | 7·4 (6·7–8·2) | 6·8 (6·1–7·5) | 0·25 |
| **Dyspnea(Q23)** | 7·1 (6·2–8·1) | 6·6 (5·5–7·5) | 0·33 | 7·2 (6·2–8·2) | 6·3 (5·3–7·3) | 0·20 | 7·3 (6·2–8·4) | 6·2 (5·1–7·2) | 0·14 | 7·4 (6·3–8·6) | 6·2 (5·1–7·2) | 0·13 |
| **Sleep disturbance(Q24)** | 14·0 (13·0–15·1) | 15·4 (14·0–16·7) | 0·12 | 14·0 (12·9–15·0) | 15·4 (14·1–16·7) | 0·09 | 13·9 (12·7–15·1) | 15·2 (13·9–16·6) | 0·16 | 14·0 (12·7–15·2) | 15·2 (13·8–16·6) | 0·19 |
| **Appetite loss(Q25)** | 18·5 (17·6–19·4) | 19·3 (18·3–20·3) | 0·23 | 18·8 (17·8–19·7) | 19·5 (18·5–20·5) | 0·33 | 19·0 (17·9–20·0) | 19·3 (18·2–20·3) | 0·70 | 19·0 (17·9–20·1) | 19·0 (17·9–20·1) | 0·97 |
| **Constipation(Q26)** | 9·0 (8·0–9·9) | 9·5 (8·5–10·5) | 0·49 | 8·9 (7·9–9·9) | 9·9 (8·9–11·0) | 0·18 | 9·0 (7·9–10·1) | 9·9 (8·8–11·1) | 0·24 | 9·4 (8·2–10·6) | 10·0 (8·9–11·1) | 0·42 |
| **Diarrhea(Q27)** | 3·7 (3·2–4·2) | 4·2 (3·7–4·7) | 0·18 | 3·8 (3·3–4·3) | 4·2 (3·7–4·7) | 0·31 | 3·8 (3·3–4·3) | 4·2 (3·7–4·8) | 0·26 | 3·8 (3·2–4·4) | 4·2 (3·6–4·7) | 0·33 |
| **Financial difficulties(Q28)** | 38·5 (37·4–39·7) | 39·4 (38·2–40·6) | 0·31 | 38·5 (37·3–39·7) | 39·7 (38·5–40·9) | 0·17 | 38·6 (37·3–39·9) | 39·7 (38·4–41·0) | 0·23 | 38·6 (37·2–38·9) | 39·9 (38·6–41·2) | 0·18 |
| **Global Health Status(Q29-30)** | 77·3 (76·8–77·8) | 76·7 (76·2–77·2) | 0·10 | 77·2 (76·7–77·7) | 76·7 (76·2–77·2) | 0·15 | 77·3 (76·7–77·9) | 76·7 (76·1–77·2) | 0·11 | 77·3 (76·6–77·9) | 76·7 (76·2–77·2) | 0·19 |

CE MRI = Contrast Enhanced Magnetic Resonance Imaging; ^18^F-FET = ^18^F-fluoroethyltyrosine ; IPTW = Inverse Probability of Treatment Weighting; EORTC = European Organisation for Research and Treatment of Cancer; QLQ-C30 = Quality of Life Questionnaire-Core 30; QLQ-BN20 = Quality of Life Questionnaire-Brain Neoplasm Module

**Tables S8. IPTW-adjusted comparison of EORTC QLQ-BN20 scores between CE MRI and ^18^F-FET PET groups at three, six, nine, and 12 months post-surgery**

|  | **3 months** | | ***p*** | **6 months** | | ***p*** | **9 months** | | ***p*** | **12 months** | | ***p*** |
| --- | --- | --- | --- | --- | --- | --- | --- | --- | --- | --- | --- | --- |
|  | **CE MRI ( Weighted n = 120·65)** | **^18^F-FET PET ( Weighted n = 113·34)** |  | **CE MRI ( Weighted n = 109·42)** | **^18^F-FET PET ( Weighted n = 105·31)** |  | **CE MRI ( Weighted n = 91·34)** | **^18^F-FET PET ( Weighted n = 98·91)** |  | **CE MRI ( Weighted n = 83·03)** | **^18^F-FET PET ( Weighted n = 91·15)** |  |
| **Future Uncertainty(Q1-4)** | 79·7 (78·6–80·7) | 80·6 (79·7–81·6) | 0·18 | 79·9 (78·8–80·9) | 80·3 (79·3–81·4) | 0·53 | 79·6 (78·4–80·8) | 80·2 (79·1–81·2) | 0·49 | 80·0 (78·8–81·3) | 80·2 (79·1–81·3) | 0·85 |
| **Visual Disorder(Q6-8)** | 9·4 (8·7–10·1) | 8·9 (8·1–9·7) | 0·31 | 9·5 (8·7–10·2) | 8·9 (8·1–9·7) | 0·30 | 9·6 (8·8–10·5) | 8·8 (7·9–9·6) | 0·17 | 9·8 (8·9–10·7) | 8·7 (7·8–9·6) | 0·10 |
| **Motor Dysfunction(Q10、15、19)** | 54·1 (51·9–56·4) | 50·9(48·2–53·7) | 0·08 | 54·0 (51·5–56·4) | 51·4 (48·6–54·3) | 0·19 | 55·2 (52·5–57·9) | 51·6 (48·6–54·7) | 0·08 | 54·9 (52·1–57·8) | 51·2 (48·1–54·4) | 0·09 |
| **Communication Deficit(Q11-13)** | 17·4 (15·6–19·2) | 19·3 (17·5–21·1) | 0·14 | 17·4 (15·5–19·3) | 18·9 (17·0–20·7) | 0·29 | 17·8 (15·8–19·9) | 18·8 (16·8–20·8) | 0·51 | 17·1 (15·0–19·3) | 18·6 (16·5–20·7) | 0·32 |
| **Social Functioning(Q14-15)** | 71·5 (69·7–73·2) | 69·3 (67·4–71·3) | 0·11 | 71·7 (69·9–73·5) | 69·8 (67·7–71·8) | 0·15 | 71·8 (69·8–73·8) | 69·0 (67·0–71·1) | 0·06 | 71·9 (69·8–74·0) | 68·8 (66·7–71·0) | 0·05 |
| **Seizures(Q9)** | 6·0 (5·3–6·7) | 5·4 (4·8–6·0) | 0·20 | 6·0 (5·2–6·8) | 5·4 (4·8–6·0) | 0·20 | 6·1 (5·3–7·0) | 5·5 (4·8–6·1) | 0·21 | 6·0 (5·1–6·9) | 5·5 (4·9–6·2) | 0·44 |
| **Drowsiness(Q14)** | 16·3 (15·2–17·3) | 17·7 (16·4–18·9) | 0·09 | 16·2 (15·1–17·2) | 17·5 (16·3–18·8) | 0·11 | 16·0 (14·8–17·1) | 17·3 (16·1–18·6) | 0·12 | 16·0 (14·8–17·2) | 17·1 (15·8–18·4) | 0·21 |
| **Hair Loss(Q16)** | 11·1 (10·4–11·8) | 11·6 (10·9–12·3) | 0·36 | 11·1 (10·4–11·9) | 11·7 (10·9–12·5) | 0·31 | 11·2 (10·4–12·0) | 11·7 (10·9–12·5) | 0·41 | 11·1 (10·2–11·9) | 11·5 (10·7–12·4) | 0·44 |
| **Itchy Skin(Q17)** | 6·9 (5·7–8·0) | 6·0 (4·8–7·2) | 0·31 | 6·6 (5·5–7·7) | 6·0 (4·8–7·3) | 0·53 | 6·9 (5·6–8·2) | 5·7 (4·5–7·0) | 0·21 | 6·6 (5·3–7·9) | 5·9 (4·5–7·2) | 0·44 |
| **Weakness of Legs(Q18)** | 18·0 (16·5–19·5) | 20·2 (18·4–21·9) | 0·07 | 18·2 (16·6–19·8) | 20·4 (18·5–22·2) | 0·09 | 19·0 (17·1–20·8) | 20·4 (18·5–22·3) | 0·28 | 18·8 (16·9–20·7) | 20·8 (18·8–22·8) | 0·16 |
| **Bladder Control(Q20)** | 6·6 (5·1–8·1) | 5·2 (4·1–6·3) | 0·13 | 6·5 (5·0–8·1) | 5·3 (4·2–6·4) | 0·22 | 6·6 (4·9–8·3) | 5·4 (4·2–6·6) | 0·24 | 6·4 (4·6–8·2) | 5·1 (3·9–6·3) | 0·22 |
| **Feel unsteady on feet(Q19)** | 18·3 (16·2–20·4) | 16·1 (14·4–17·8) | 0·12 | 18·5 (16·2–20·7) | 16·2 (14·5–18·0) | 0·13 | 18·3 (15·8–20·7) | 16·3 (14·4–18·1) | 0·20 | 18·1 (15·5–20·7) | 15·8 (13·9–17·7) | 0·16 |

CE MRI = Contrast Enhanced Magnetic Resonance Imaging; ^18^F-FET = ^18^F-fluoroethyltyrosine ; IPTW = Inverse Probability of Treatment Weighting; EORTC = European Organisation for Research and Treatment of Cancer; QLQ-C30 = Quality of Life Questionnaire-Core 30; QLQ-BN20 = Quality of Life Questionnaire-Brain Neoplasm Module
