# Supplementary material for: A New Fluorine-18-Fluoroethyltyrosine Positron-Emission-Tomography/Magnetic-Resonance-Based Tumor Resection Plan Improved the Prognosis of Glioblastoma Patients. A Multicenter Validated Study
Source: Research (Wash D C). 2026 Aug 4;9:1335. doi: 10.34133/research.1335 (PMC13434045; doi:10.34133/research.1335)
Supplement: Supplementary file 1 [file research.1335.f1.zip › Supplementary figure1.docx]

**Supplementary Fig. 1. KM survival analysis after IPTW comparing the ^18^F-FET PET and CE MR groups. Shaded areas represent 95% CIs. Numbers at risk at each time point are listed below the x-axis.**

(A) KM curve for OS. The ^18^F-FET PET group showed a significantly longer median OS of 19.7 months compared to 16.0 months in the CE MRI group (p = 0.01). (B) KM curve for PFS. The PET group had a significantly longer median PFS of 12.5 months, whereas the MRI group had a median PFS of 9.3 months (p = 0.01). KM = Kaplan-Meier; CE MRI, contrast enhanced MRI; ^18^F-FET PET = 18F-fluoroethyltyrosine PET; OS = overall survival; PFS = progression free survival; CIs = confidence intervals.
